# Developing high-quality value-added cereals for organic systems in the US Upper Midwest: hard red winter wheat (*Triticum aestivum* L.) breeding

**DOI:** 10.1007/s00122-022-04112-0

**Published:** 2022-05-28

**Authors:** Pablo Sandro, Lisa Kissing Kucek, Mark E. Sorrells, Julie C. Dawson, Lucia Gutierrez

**Affiliations:** 1 Department of Agronomy, University of Wisconsin-Madison, Madison, WI 53706 USA; 2 Dairy Forage Research Center, USDA-ARS, Madison, WI 53706 USA; 3 Plant Breeding, and Genetics Section, School of Integrative Plant Sciences, Cornell University, Ithaca, NY 14853 USA; 4 Department of Horticulture, University of Wisconsin-Madison, Madison, WI 53706 USA

## Abstract

**Abstract:**

There is an increased demand for food-grade grains grown sustainably. Hard red winter wheat has comparative advantages for organic farm rotations due to fall soil cover, weed competition, and grain yields. However, limitations of currently available cultivars such as poor disease resistance, winter hardiness, and baking quality, challenges its adoption and use. Our goal was to develop a participatory hard red winter wheat breeding program for the US Upper Midwest involving farmers, millers, and bakers. Specifically, our goals include (1) an evaluation of genotype-by-environment interaction (GEI) and genotypic stability for both agronomic and quality traits, and (2) the development of on-farm trials as well as baking and sensory evaluations of genotypes to include farmers, millers, and bakers’ perspectives in the breeding process. Selection in early generations for diseases and protein content was followed by multi-environment evaluations for agronomic, disease, and quality traits in three locations during five years, on-farm evaluations, baking trials, and sensory evaluations. GEI was substantial for most traits, but no repeatable environmental conditions were significant contributors to GEI making selection for stability a critical trait. Breeding lines had similar performance in on-station and on-farm trials compared to commercial checks, but some breeding lines were more stable than the checks for agronomic, quality traits, and baking performance. These results suggest that stable lines can be developed using a participatory breeding approach under organic management. Crop improvement explicitly targeting sustainable agriculture practices for selection with farm to table participatory perspectives are critical to achieve long-term sustainable crop production.

**Key message:**

We describe a hard red winter wheat breeding program focused on developing genotypes adapted to organic systems in the US Upper Midwest for high-end artisan baking quality using participatory approaches.

**Supplementary Information:**

The online version contains supplementary material available at 10.1007/s00122-022-04112-0.

## Introduction

### Breeding for sustainable systems: potentials for organic hard red winter wheat

Plant breeding has been highly successful in improving crops for serving humans food, fiber, and fuel requirements by focusing on traits such as yield, quality, and disease resistance among others (Bernardo 2010; Duvick 2003). There is currently a need to achieve sustainable intensification by expanding the food, feed, and fuel goals to include soil, water, and biodiversity targets for agriculture (Heaton et al. 2013; Runck et al. 2014). Because of their role in creating new crops, plant breeders play a crucial role in the development of sustainable agricultural systems for the future (Brummer et al. 2011). Some general breeding strategies have included selection for lower input requirements that may decrease fossil fuel use, water contamination, and production cost (Tilman 1999; Robertson and Swinton 2005; Dawson et al. 2008) or adaptation to stress (Araus et al. 2008; Cattivelli et al. 2008; Jacobs 2007; Santini et al. 2007). Other strategies include breeding for highly diverse cropping systems (Ceccarelli et al. 2010; Liebman 2012) and regional breeding strategies to withstand variable and or changing environments. Breeders have also focused on selection for local adaptation (Ceccarelli and Grando 2007) and cropping systems (Cook 2006) to improve performance under specific environments while providing ecosystem services. Finally, breeding for winter-annual or perennial crops to provide continuous living cover on land that would not otherwise be cropped over the winter leads to strategic spatio-temporal utilization of resources and improved environmental outcomes (Runck et al. 2014; Schulte et al. 2006; Heaton et al. 2013), selection for emerging agricultural systems such as natural ecosystems (Glover et al. 2010) or organic agriculture (Dawson et al 2008; 2011; Lammerts van Bueren and Myers 2012; Wolfe et al. 2008) can also improve agricultural sustainability. These strategies have been successfully implemented in a diverse set of crops and cropping systems including cereals such as wheat (*Triticum aestivum* L.).

Wheat is the most important food grain produced in the USA, with production over 51 million metric tons (FAOSTATS 2020), and the USA is among the top three exporters of wheat in the world. However, wheat differs from other major crops grown in the USA because the majority of the US commercial wheat varieties are developed by public plant breeding programs (Wheat CAP 2018), contributing billions in production value (USDA/NASS 2021). Furthermore, in the predominant wheat-producing regions of the USA, growers are supported by a commodity grain system and industrial baking markets, with most breeders developing varieties adapted to conventional systems (Tilley et al. 2012; Kiszonas and Morris 2018). However, there has been a very limited effort in developing wheat varieties adapted to organic systems in the USA.

In many regions of the USA, the demand for local and organic products is growing (Green et al. 2017; Rana and Paul 2017), while organic production is one of the fastest growing sectors in agriculture (Matlock 2021). Specifically, land in organic wheat production increased 15% from 2015 to 2019 (Matlock 2021) and production is expected to continue growing since revenue and the number of organic farms has been growing in recent years in the USA (15% 2017–2019) and particularly in the US Upper Midwest (10% 2017–2019) (USDA/NASS 2019). Although most cereal grains in the US Upper Midwest are grown for feed, food-grade grains present a high-value and more consistent market outside of the commodity wheat system. Local grain markets are also growing, and both organic and local markets are responding to strong consumer demand for artisanal breads such as whole grain naturally leavened (sourdough). Expanding the production of food-grade grains for artisanal products also supports an increase in whole grain consumption with its associated health benefits (Mellen et al. 2008; Jonnalagadda et al. 2011; Reynolds et al. 2019). However, few breeding programs are dedicated to developing varieties for whole grain end-uses or sourdough, resulting in a lack of evaluation and selection strategies as well as limited availability of varieties with suitable end-use quality (Ross 2018; Krill-Brown et al. 2019). Therefore, breeding programs developing hard wheat varieties adapted to regional climates and organic production with good quality for artisanal products are necessary and will provide farmers with greater opportunities to reach high-value markets.

Breeding targets for hard (bread) wheat generally include grain yield, winter hardiness, protein concentration, resistance to pre-harvest sprouting (for baking quality) and resistance to Fusarium head blight (*Fusarium graminearum* L., FHB) and foliar diseases. However, cultivars selected under conventional systems may not be suitable for organic production because genotype by management performance rank changes have been observed between organic and conventional systems for grain yield and protein content in cereals in general (Przystalski et al. 2008; Wolfe et al. 2008) and in wheat in particular (Reid et al. 2009; Hildermann et al. 2009). Moreover, this strong interaction between genotype and management system, and high heritability for these traits in organic trials, makes direct selection under organic conditions more effective (Murphy et al. 2007; Przystalski et al. 2008). A survey and interviews with organic wheat farmers identified traits where more research and development are needed for organic bread wheat production (Kucek 2017). High protein with artisanal baking quality and flavor, disease resistance including resistance to FHB and leaf diseases, winter hardiness, weed competitive ability, and overall good agronomic performance were identified as the key target traits for farmers (Kucek 2017). Discussions with bakers and millers have identified stability for quality parameters and good technical performance in whole grain and sourdough products as key traits. Stability is particularly important in regional grains systems as less blending is possible for grain with different quality parameters from different production regions to buffer yearly fluctuations in protein levels, falling number or disease incidence.

### Breeding for climate-resilient and stable crops: participatory plant breeding as an alternative

In addition to meeting the target traits for organic systems, it is important to consistently obtain high-quality grain across locations and years. There are two levels where stability is important: temporal and spatial. Organic farms tend to be more diverse from farm-to-farm and field-to-field than conventional farms (Shennan et al. 2017; Knapp and van der Heijden 2018) with additional changes in genotypic rankings from year to year (Kucek et al., 2019) and would therefore benefit from both spatial and temporal stability. Furthermore, crop yield losses resulting from extreme temperatures, recurring droughts, and erratic rainfall patterns due to climate change are expected to have global impact (Wassman et al. 2009). The goal of breeding for climate-resilient crops is to achieve crop yield not limited by multiple challenges related to abiotic stress, such as drought or heat stress (Bhatta et al. 2018; Picasso et al. 2019), and biotic stresses, such as diseases, pests, and weeds (Ceccarelli et al. 2010; Malosetti et al. 2013; Wani et al. 2018). Therefore, breeding efforts should focus on continuing to identify genotypes with high yield potential while reducing the yield gap under sub-optimal growing conditions due to extreme weather events and climate change (Araus et al. 2008; Fischer and Edmeades 2010; Pennacchi et al. 2019). The increasing frequency of extreme weather events means that the environmental conditions faced by new genotypes may be outside of the range of historic variation in a target region. Therefore, breeders need to select for varieties with broad adaptation to larger areas and high yield stability that would decrease climate-induced risk and build resilience (Langridge et al. 2021). Longer-term stability may also be achieved with high levels of genetic diversity. Within variety, genetic diversity can be achieved either by maintaining some level of residual heterozygosity or from lines being fixed for different alleles after the line was derived early in the breeding program (Dawson and Goldringer 2012). Alternatively, genetic diversity can be achieved at the field level by growing multiple varieties in a field (Weedon and Finckh 2019; Wolfe and Ceccarelli 2020).

One way for breeding programs to address the heterogeneity of target environments and emerging markets is to work directly with farmers, bakers, and other expert professionals. Participatory plant breeding, where farmers and other stakeholders such as bakers, millers, seed producers, and consumers are involved in the breeding process, can take many shapes and may have different levels of involvement (Lammerts van Bueren and Myers 2012). One of the aspects that participatory plant breeding can address is the gap between performance at the experimental stations and in farmers’ fields (Simmonds 1980, 1991; Annicchiarico 2002). There are multiple reasons why this gap occurs, but a primary cause is that in some cases, selection environments are not representative of production environments (Ceccarelli and Grando 2007).

Involving farmers and growers in the decision-making process for the selection and advancement of candidate lines would increase the adoption of new variety releases (Annicchiarico 2002), provide helpful information for breeding decisions (Ashby 2009), have industry-ready varieties for release, and provide a more diverse set of environmental evaluations (Ceccarelli and Grando 2007). Participatory plant breeding could include other stakeholders involved in the wheat production from the farm to the table such as millers, bakers, and consumers in order to increase the success of varieties (Dawson et al. 2011). Participatory bread wheat breeding programs for organic systems and artisanal baking exist in Europe (France and Italy), Canada, and in Washington State (USA). Many of these programs share methods and follow similar selection protocols. This includes using organic trials and farmer input to select parents for crosses, decentralizing selection and testing starting as early as possible, often in the F_3_ or F_4_ generation, and involving farmers and bakers in the evaluation of breeding lines on research stations, farms, and bakeries. These programs were reviewed by Colley et al. (2021).

The objective of this paper was to illustrate the development of a small breeding program for bread wheat varieties targeted to organic systems and artisan baking in the US Upper Midwest. We have involved farmers, millers, and bakers in the decision process for advancing breeding lines. Specifically, our goals include: (1) description of the breeding process from initial crosses to end-product, (2) analysis of the genotype-by-environment interaction (GEI) for agronomic and quality performance, including the study of stability indicators for transient year-to-year GEI, (3) a comparison of on-station and on-farm performance for advanced breeding lines, and the incorporation of on-farm evaluation and farmers input in the decision process, and (4) development of baking trials with bakers input to evaluate artisan whole grain baking quality in advanced breeding lines.

## Materials and methods

### Population development

**Fig. 1:**
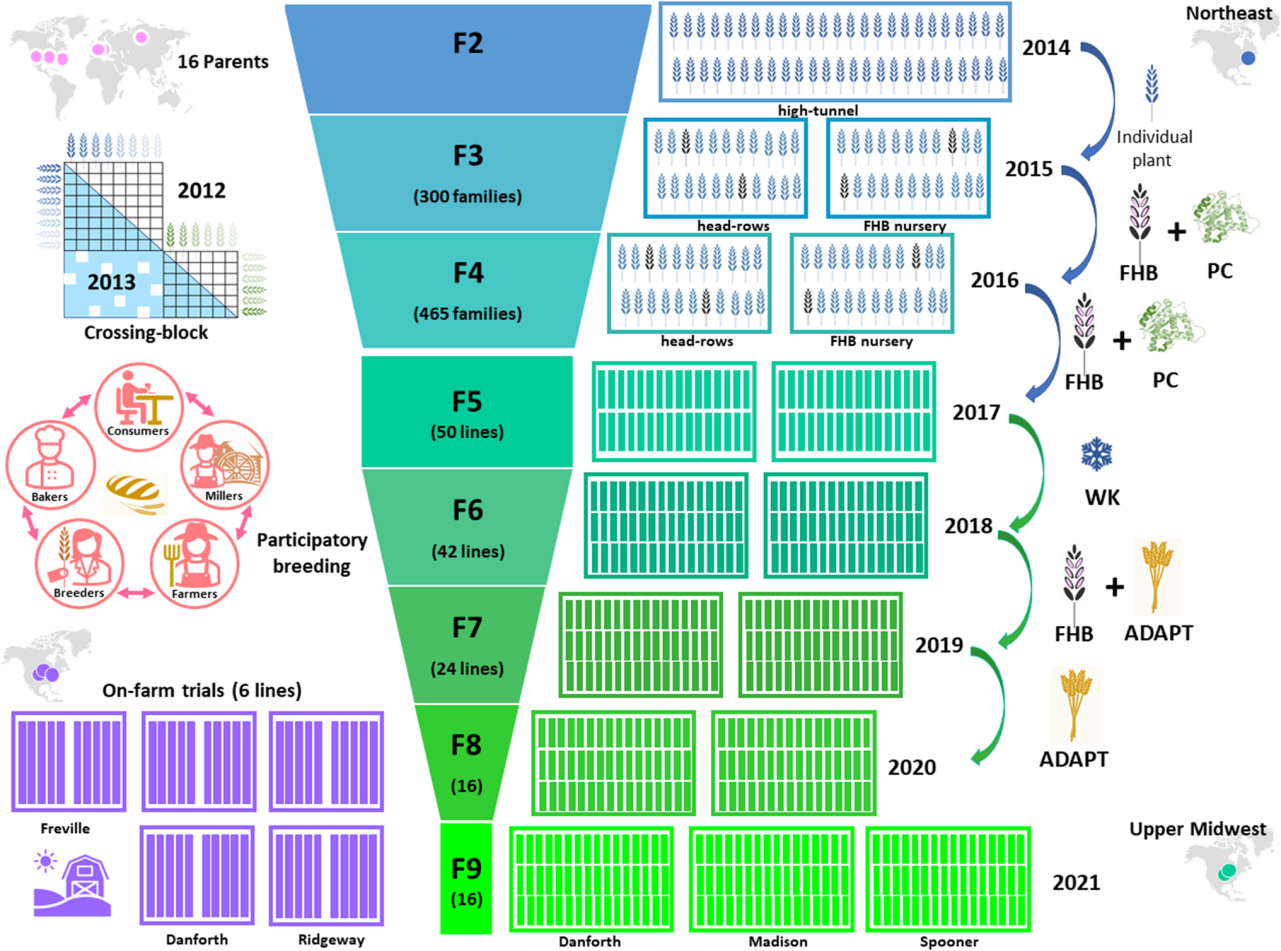
Characterization of the organic hard winter wheat breeding program. Sixteen parents were used in a partial diallel crossing block to generate the initial breeding population, and families were selected initially based on Fusarium head blight and protein content in the Northeast. At the F5 generation, lines were evaluated in the US Upper Midwest in three on-station locations (Madison, WI; Spooner, WI; and Danforth, IL) in five years (2017–2021). Advanced lines were evaluated in three on-farm locations (Freeville, NY; Danforth, IL; and Ridgeway, WI). Participatory breeding strategies were used to include farmers, millers, bakers, and consumers in the decision to advance and release breeding lines

Sixteen parents of hard winter wheat tested in organic systems in the USA and Europe were selected based on their bread quality attributes, adaptation to organic conditions, and resistance to FHB and other diseases and were used as progenitors of the breeding program (see Fig. 1 for a description of the breeding program). Eight parents were crossed in a partial diallel in 2012 in a greenhouse at Cornell University (Ithaca, NY). A second crossing block was conducted in 2013 to add eight additional parents to the partial diallel based on results from multi-location organic winter wheat trials. Between one and 20 successful F_1_ progeny per cross were self-pollinated. F_2_ plants were grown in a high tunnel from October 2013 to June 2014 and up to 30 individual plants per original cross were selected. A total of 300 F_2:3_ derived families were evaluated with 12 parents used as checks from October 2014 to July 2015 in 1 m head-rows in two trials: an agronomic trial at the Cornell University Homer C. Thompson Vegetable Research Farm in Freeville, New York (42.5° N, 76.3° W) under certified organic conditions and an FHB nursery at the Caldwell Research Farm in Ithaca New York (42.5° N, 76.3° W). Head-rows in the certified organic location were evaluated for lodging, winter survival, grain yield, and test weight. In the FHB nursery, the lines were inoculated with FHB to cover early, mid- and late flowering lines on June 2, 5, 9, 11 and 16, 2015 (Zadoks 61, 65, and 69). Plants were inoculated by spaying a solution containing an inoculum dilution at 1 × 10^–5^ conidia per ml. The FHB inoculum was prepared following the protocol reported in Fulcher and Bergstrom (2020). Head-rows were irrigated with an intermittent mist for 20 s at 5-min intervals from 8:00 AM to 8:00 PM between heading (Zadocks 59) and milky grain stages (Zadocks 77). Head-rows were scored for FHB incidence and severity at the soft dough stage (Zadock 83), about 24 days after flowering following Fulcher et al. (2021). Briefly, twenty spikes in each head-row were scored from 0 to 5 for FHB where 0 = no infected spikelet, 1 = one spikelet infected, 2 = two spikelets infected, 3 = up to half of the spikelets in the spike infected, 4 = more than half of the spikelets in the spike infected, and 5 = the entire spike is dead. The incidence of FHB was calculated as the percent of nonzero scores among the 20 spikes scored, FHB severity was calculated as the average percentage of infected spikelets, and the FHB index was the product of the incidence and severity expressed as a percentage. From 115 F_2:3_ families that scored an FHB index less than 15, ten individual plants were selected at Freeville, NY and eight seeds from each plant were evaluated for protein content using a near infrared spectroscopy single-seed analyzer following Carlson et al. (2019). F_2:3_ families with mean protein content above the 50% percentile were selected. These 465 F_3:4_ families were evaluated from October 2015 to July 2016 in 1 m head-rows in two trials: an agronomic trial and a FHB nursery. The agronomic trial was conducted at the Freeville location in an augmented design using three check lines replicated 10 times throughout the nursery to correct for spatial variability. Families were evaluated for lodging, winter survival, grain yield, and test weight. The FHB nursery was conducted in Caldwell, but due to seed limitations for some families, only 406 F_3:4_ families were grown in the FHB nursery. An augmented design with two check lines replicated 10 times was used to correct for spatial variability. F_3:4_ families were selected based on an index incorporating grain yield, leaf disease, and FHB index and 98 families were selected. Eight seeds from each selected family were evaluated for protein content using a near infrared spectroscopy single-seed analyzer following Carlson et al. (2019), and the 50 families with the highest mean protein content were advanced to F_5_.

### On-station phenotypic evaluation

The 50 selected advanced inbred lines (i.e., F_5_ families derived from F_3:4_ families) and three organic artisan milling and baking industry checks (‘Red Fife,’ ‘Arapahoe,’ and ‘Warthog’) were evaluated between 2017 and 2021 at the West Madison Agricultural Research Station in Verona, Wisconsin (Madison, 43°04′22.6′′ N 89°32′46.8′′ W), and at Janie’s Farm in Danforth, Illinois (Danforth, 40°50′30.2′′ N 87°58′10.1′′ W). A third location was used during the 2021 year at Spooner Agricultural Research Station in Spooner, Wisconsin (Spooner, 45°49′21.0′′ N 91°52′35.3′′ W). Trials were planted as close as possible to ideal planting dates for each location ranging from September 25^th^ to October 22nd. Planting dates must fall between late September and early October, as earlier plantings are susceptible to aphid feeding that may transmit barley yellow dwarf virus, but later plantings may not have enough growing degrees days remaining to support seedling establishment and growth required for winter survival (Conley et al. 2015). All trials were managed following organic practices on certified organic land, except for the Spooner location, which has been managed according to organic standards since 2018 but is not certified. ‘Red Fife’ is an historic facultative variety originally selected in Ontario, Canada, that is popular for baking because of its flavor and its inclusion in the Slow Food Ark of Taste (Ark of Taste 2014). ‘Red Fife’ was a standard for the Canadian baking and milling industries in the mid-late 1900s. ‘Arapahoe’ is a modern hard red winter wheat variety with very good winter hardiness and baking quality released by the University of Nebraska in 1989 (Baenziger et al. 1989). ‘Arapahoe’ has high protein levels and test weight and is resistant to stem rust and moderately resistant to leaf rust. ‘Warthog’ is a modern hard red winter wheat variety released in Canada in 2001 (Registration #5390, Thompsons Limited). ‘Warthog’ has been widely grown by farmers in the US Upper Midwest and has acceptable baking quality.

Experiments at Madison followed a four-year rotation: 3 years of alfalfa followed by one year of cereals. Management of the experiments included mechanical weeding using mowers in alleys and a cultivator between plots (2019 and 2021), hand weeding inside plots (intense weeding in 2017 and 2018, but minimal weeding in 2019 and 2021), hand rogueing off-types to maintain genetic purity and to avoid cross-contamination (i.e., one or two plants per plot), and frost-seeded red clover planted in the winter to maintain soil cover. The Madison 2017 experiment was a randomized complete block design (RCBD) with two replications. The Madison 2018, 2019, and 2020 experiments were augmented alpha designs with three complete replications. The Madison 2021 experiment was a RCBD with three replications. The Madison 2017 experiment was evaluated in 1.2 × 1.85 m (2.22 m^2^) plots, while the 2018–2021 experiments were evaluated in 1.52 × 3.20 m (4.86 m^2^) plots. Planting was on October 3, 2016, September 28, 2017, September 28, 2018, September 25, 2019, and September 25, 2020. Planting density was 134 kg ha^−1^ for the 2017 experiments and 157 kg ha^−1^ for the 2018–2021 experiments. Clover was frost-seeded on March 3, 2017, February 16, 2018, February 3, 2019, February 16, 2020, and February 26, 2021. Harvest dates were July 17, 2017, July 18, 2018, July 18, 2019, July 17, 2020, and July 10, 2021. All experiments were planted with an inter-column spacing of 30 cm and an inter-row spacing of 2.4 m.

The 2021 experiment at Spooner was a RCBD with three replications evaluated in 1.52 × 3.65 m (5.54 m^2^) plots. Planting was on September 29, 2020, at a planting density of 157 kg ha^−1^, while harvest date was July 21, 2021.

Experiments at Danforth consisted of a RCBD with two (2017) or three (2018–2021) replications evaluated in 1.4 × 2.4 m (3.36 m^2^) plots with a planting density of 117 kg ha^−1^, an inter-column spacing of 23.3 cm and an inter-row spacing of 30 cm. Planting was on October 22, 2016, October 3, 2017, September 22, 2018, October 3, 2019, and September 9, 2020, for the 2017–2021 growing seasons. Harvest date was July 18, 2017, June 26, 2018, July 11, 2019, July 7, 2020, and July 8, 2021.

All genotypes were evaluated for agronomic traits including winter survival, plant height, lodging, heading date and grain yield; disease traits including FHB, leaf rust, barley yellow dwarf virus, and powdery mildew; and for grain quality traits including test weight, falling number, ash, and protein content. Winter survival was estimated as the percent of plants that were still green after the snow melts in the spring in early April. Plant height was measured during the grain filling stage (Zadoks 73–83) as the height (in cm) of an average plant in the plot from the ground to the tip of the spike not including awns. Lodging was estimated after the grain filling stage (Zadoks 83–92) as the percent of plants in the plot that had an angle of 45° or lower with the horizontal. Heading date was recorded on the date when 50% of the tillers in a plot had spikes completely emerged from the flag leaf sheath (Zadoks 60). Grain yield was evaluated as the weight of each plot harvested at maturity (Zadoks 92) and expressed in kg ha^−1^ obtained by harvesting and threshing with a self-propelled Wintersteiger Masterplot experimental plot combine harvester, drying the grain, and cleaning the grain with a Pfeuffer Sample MLN grain cleaner. Grain yield was corrected to a kernel moisture level of 12%. FHB was evaluated on a 1–9 scale using a combined score between incidence and severity after flowering (Zadoks 83) using natural infection. Leaf rust and powdery mildew were scored on a 1–9 scale similar to FHB based on natural infection in years where the disease was detected. Incidence of barley yellow dwarf virus was evaluated based on natural infection as the percentage of plants affected by the disease in years where the disease was present. All agronomic and disease traits were recorded in Madison. Grain yield, plant height, and heading date were recorded in Danforth. Grain yield, plant height, and heading date were recorded in Spooner.

Test weight was measured as the weight of 500 mL of dry and clean grains using a Cox funnel and following the Canadian Grain Commission official grain grading guide (Grain Commission). A subsample of 500 g of grain from each plot from the 2019, 2020, and 2021 experiments was sent to the Integrated Bioprocessing Research Laboratory of the College of Agricultural, Consumer and Environmental Sciences at the University of Illinois at Urbana-Champaign for quality processing. Grain samples were analyzed for grain protein content, falling number, and ash content. Protein and ash content were evaluated with a Perten Inframatic 9500 NIR (near infrared) grain analyzer and expressed in percent values. Falling number was evaluated with a Perten Falling Number® system that measures alpha-amylase enzyme activity and structural integrity of the starch and is expressed in seconds. Grain quality traits were evaluated on samples from Madison and Danforth.

### Statistical analysis of on-station experiments

#### On-station genotypic means

Empirical best linear unbiased estimates (BLUEs) of each agronomic and quality trait for each genotype in each location and year (i.e., environment) were estimated using plot-level information from all genotypes planted at a given location and year (i.e., environment) and corrected for experimental design and spatial variation with the following linear mixed model:

1 $$\underline{{y_{ijkl} }} = \mu + G_{i} + \underline{{\beta_{j} }} + \underline{{R_{k} }} + \underline{{C_{l} }} + \underline{{\varepsilon_{ijkl} }}$$

where *y*_*ijkl*_ is the plot-level observation, *µ* is the overall mean, *G*_*i*_ is the effect of the ith genotype, *β*_*j*_ is the effect of the *j*th block with *β*_*j*_ ~ *N*(0, *σ*^2^_*β*_), *R*_*k*_ is the effect of the *k*th row with *R*_*k*_ ~ *N*(0, *σ*^2^_*R*_), *C*_l_ is the effect of the *l*th column with *C*_l_ ~ *N*(0, *σ*^2^_*C*_), and *ε*_*ijkl*_ is the residual term with *ε*_*ijkl*_ ~ *N*(0, *σ*^2^_*ε*_), with the covariance among random effects equal to zero and *σ*^2^_*β*_, *σ*^2^_*R*_, *σ*^2^_*C*_, and *σ*^2^_*ε*_, being the variance components of blocks, row, column, and residual error, respectively. Row and column effects were used as a post-blocking control of spatial variation and were only considered when their inclusion improved the model fit (see supplementary files 1–5). An alpha design was used in some trials with incomplete blocks following rows, and therefore, the same model was used for those trials. This analysis was performed in R statistical software (R. Core Team 2013) fitting the *lmer* function of the *lme4* package (Bates et al. 2007). Genotypic BLUEs were obtained using the *emmeans* function of the *emmeans* package (Lenth 2021) in R statistical software. This model was used for all agronomic and quality traits in each environment (i.e., combination of location and year).

#### Variance components estimation

The following random effects model was used to estimate variance components for genotype, location, year, their interactions (i.e., location by year, genotype by year, genotype by location, and genotype by location by year) and the residual error for grain yield, protein content, falling number, and ash content:

2 $$\underline{{y_{ijkl} }} = \mu + \underline{{G_{i} }} + \underline{{L_{j} }} + \underline{{A_{k} }} + \underline{{LA_{jk} }} + \underline{{\beta_{{l\left( {jk} \right)}} }} + \underline{{GL_{ij} }} + \underline{{GA_{ik} }} + \underline{{GLA_{ijk} }} + \underline{{\varepsilon_{ijkl} }}$$

where *y*_*ijkl*_ is the plot-level observation, *µ* is the overall mean, *G*_*i*_ is the effect of the *i*th genotype with *G*_*i*_ ~ *N*(0, *σ*^2^_*G*_), $L_{j}$ is the effect of the *j*th location with *L*_*j*_ ~ *N*(0, *σ*^2^_*L*_), *A*_*k*_ is the effect of the *k*th year with *A*_*k*_ ~ *N*(0, *σ*^2^_*A*_), $LA_{jk}$ is the location-by-year interaction with *LA*_*jk*_ ~ *N*(0, *σ*^2^_*LA*_), *β *_*i(jk)*_ is the effect of the *j*th block with *β*_*i(jk)*_~ N(0, *σ*^2^_*β*_), $GL_{ij}$ is the genotype-by-location interaction with *GL*_*ij*_ ~ *N*(0, *σ*^2^_*GL*_), $GA_{ik}$ is the genotype-by-year interaction with *GA*_*ik*_ ~ *N*(0, *σ*^2^_*GA*_), $GLA_{ijk}$ is the genotype-by-location-by-year interaction with *GLA*_*ijk*_ ~ *N*(0, *σ*^2^_*GLA*_), and *ε*_*ijkl*_ is the residual term with *ε*_*ijkl*_ ~ *N*(0, *σ*^2^_*ε*_), with the covariance among random effects equal to zero and *σ*^2^_*G*_, *σ*^2^_*L*_, *σ*^2^_*A*_, *σ*^2^_*LA*_, *σ*^2^_*β*_, *σ*^2^_*GL*_, *σ*^2^_*GA*_, *σ*^2^_*GLA*_, and *σ*^2^_*ε*_, being the genotypic, location, year, location-by-year, block, genotype-by-location, genotype-by-year, genotype-by-location-by-year, and residual error variance components. The variance components were then expressed as a proportion of the total genetic variance (*σ*^2^_*G*_ + *σ*^2^_*GL*_ + *σ*^2^_*GA*_ + *σ*^2^_*GLA*_).

#### Heritability

Heritability for each trait in each environment was calculated ad hoc following Piepho (2019) based on Holland et al. (2003) using harmonic means:

3 $$H^{2} = \frac{{\sigma_{g}^{2} }}{{\sigma_{g}^{2} + \frac{{\sigma_{ga}^{2} }}{{\overline{n}_{a} }} + \frac{{\sigma_{gl}^{2} }}{{\overline{n}_{l} }} + \frac{{\sigma_{gal}^{2} }}{{\overline{n}_{al} }} + \frac{{\sigma_{e}^{2} }}{{\overline{n}_{alr} }}}}$$

where *H*^*2*^ is the estimate of the heritability, $\sigma_{g}^{2}$_,_ $\sigma_{ga}^{2}$_,_ $\sigma_{gl}^{2}$_,_ $\sigma_{gal}^{2}$_,_ and $\sigma_{e}^{2}$ are the variance component estimates of genotype, genotype by year, genotype by location, genotype by year by location, and the residual error, respectively, from model (2), and $\overline{n}_{a}$, $\overline{n}_{l}$, $\overline{n}_{al}$, and $\overline{n}_{alr}$ the harmonic means of the number of years, locations, location-year, and location-year-replications. Variance components and heritabilities were also estimated from models (2) and (3) for each location removing the location components and for each environment removing the location-year components.

*GGE biplots.* BLUEs from model (1) were used to graphically represent the genotype and genotype-by-environment effects through a biplot (GGE biplot, Wickham 2016) using the package *gge* (Laffont et al. 2013) from the R statistical program (R. Core Team 2013).

#### Finlay–Wilkinson regression

Stability for grain yield was estimated with the Finlay and Wilkinson (1963) analysis (hereafter FW) using the following model:

4 $$\underline{{y_{ij} }} = \mu + G_{i} + E_{j} + \beta_{i} E_{j} + \underline{{\varepsilon_{ij} }}$$

where *y*_*ij*_ is the BLUE of the *i*th genotype in the *j*th environment (i.e., combination of location and year) estimated from Eq. (1), *µ* is the overall mean, *G*_*i*_ is the effect of the *i*th genotype, *E*_*j*_ is the effect of the *j*th environment, *β*_*i*_ is the FW regression coefficient of the *i*th genotypic performance over the environmental mean, also called sensitivity, and $\varepsilon_{ij}$ is the residual genotype-by-environment interaction not explained by the FW regression model, where $\varepsilon_{ij}$ ~ *N*(0, *σ*_*ε*_^2^). Genotypes with sensitivity values closer to zero have static stability, while genotypes with sensitivity close to one have dynamic stability, and genotypes with higher sensitivity have low stability. The *R*^2^ coefficient representing the lack of fit of the FW regression for each genotype was estimated with the same model. The FW regression models were run on R statistical program with basic regression functions (R. Core Team 2013).

#### Wricke’s ecovalence stability coefficient

Wricke’s ecovalence stability was estimated for grain quality, protein content, falling number, and ash content using the following model (Wricke 1962):

5 $$W_{i} = \mathop \sum \limits_{j} (\hat{G}_{ij} - \hat{G}_{i} - \hat{E}_{j} - \hat{\mu })^{2}$$

where $W_{i}$ is Wricke’s ecovalence stability coefficient $\hat{G}_{ij}$ is the BLUE of the *i*th genotype in the *j*th environment (combination of location and year) estimated from Eq. (1), $\hat{G}_{i}$ is the genotypic mean of the *i*th genotype across environments, $\hat{E}_{j}$ is the environmental mean of the jth environment across all genotypes, and $\hat{\mu }$ it is the overall mean. Wricke’s ecovalence stability coefficient represents the volatility of genotypes to changes in the environment; a genotype with no genotype-by-environment interaction will show a *W* value of zero. Wricke’s ecovalence was estimated using the *StatGxE* package (van Rossum et al. 2021) in R statistical program (R. Core Team 2013).

### Selection of advanced breeding lines

**Fig. 2:**
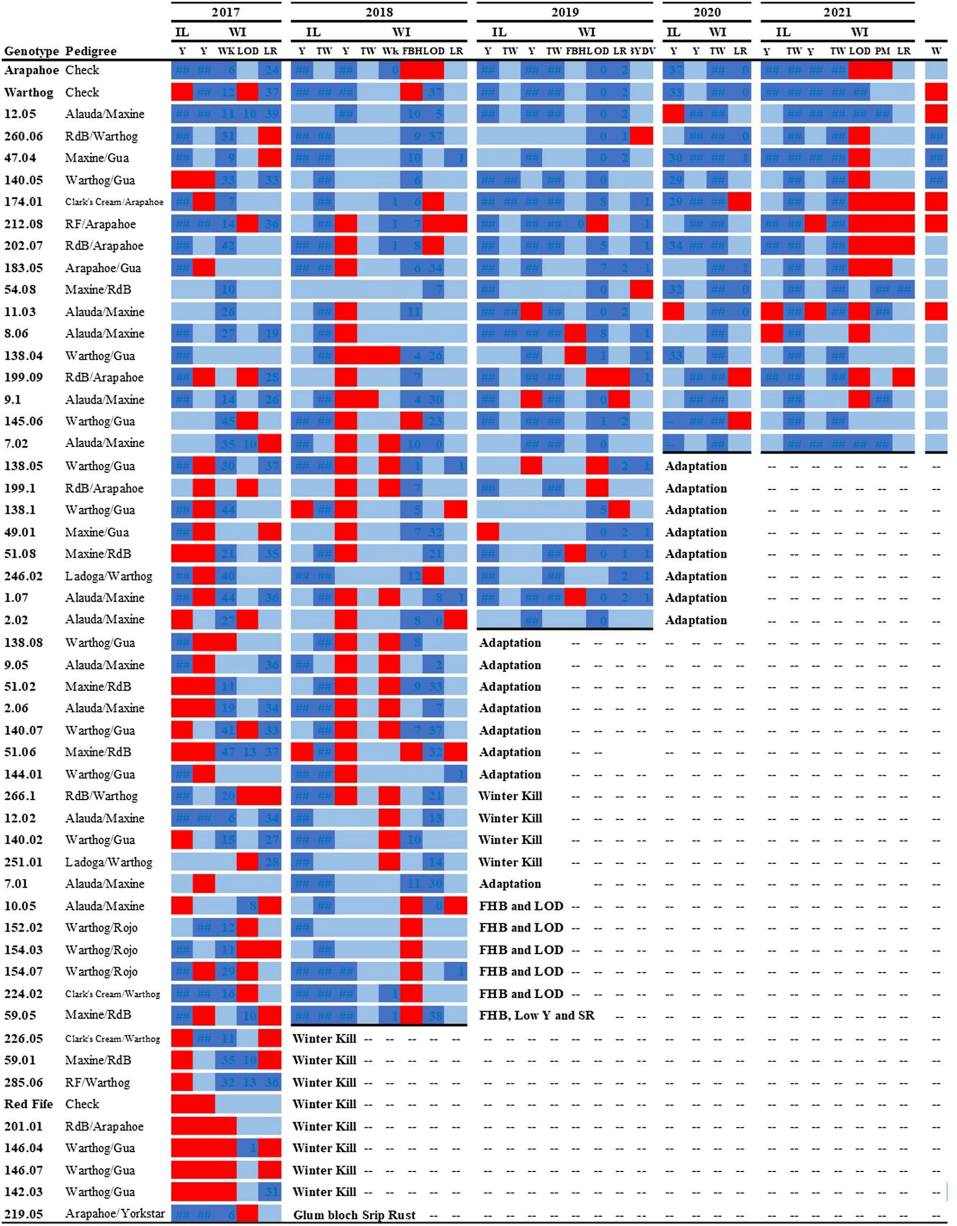
Characterization of the advanced breeding line selection process in the hard red winter wheat breeding program for artisan baked whole grain. The heatmap is a qualitative characterization of trait performance categories with blue being good performer, light blue an average, and red a bad performer for each trait in each location (IL, Danforth, Illinois; WI, Madison, Wisconsin) and year (2017–2021). Grain yield (Y) and Wricke’s ecovalence stability coefficient (W) relative performance was color-coded in relation to the overall mean performance across all environments. Winter survival (WK), lodging (LOD), leaf rust severity (LR), test weight (TW), fusarium head blight severity (FHB), powdery mildew (PM), and barley yellow dwarf virus severity (BYDV) were color-coded in absolute values. The main driver for discontinuation of early lines was indicated at the right of the heatmap of their last evaluation in the program

Advanced breeding lines were selected for grain yield, test weight, FHB and other diseases, winter survival, and protein content. The selection was applied over the population reducing the number of genotypes each year from 50 to 42 in 2018, to 24 in 2019, and finally, to 16 in 2020 (Fig. 2). The ‘Red Fife’ check was also discarded after the first year of evaluation because it did not survive the first winter and was not included in the multi-year analysis. The BLUEs for all traits, field notes, and input from farmers and bakers, described below, were used in the process of selection conducted each year based on the data from current and previous years. Winter survival was the trait with the highest selection pressure during the years 2017 and 2018. Lines that did not survive or showed more than 70% winterkill were discontinued. High selection intensity for absence of FHB symptoms was applied in all years with high natural infection. Other traits such as lodging and disease prevalence were criteria used to discard additional lines each year (Fig. 2; supplementary files 1–5). Finally, pedigree information was considered in the selection process to avoid shrinking the genetic diversity early in the process. Among and within original F_1_ cross, selection was performed through the years, but at least one genotype per cross was always advanced unless it consistently underperformed.

### Participatory on-farm trials

#### On-farm experiments

Six breeding lines and one commercial check were evaluated in on-farm trials on three farms in three states in the USA: Harold Wilken, Janie’s Farm, Danforth, IL (40°50′30.2′′ N 87°58′10.1′′ W), John and Halee Wepking and Paul Bickford, Meadowlark Organics, Ridgeway, WI (42°59′25.2′′ N 90°00′43.0′′ W), and the Freeville Organic Research Farm, Freeville, New York (Freeville, 42°31′06.7′′ N 76°20′04.6′′ W). The two farms in the Upper Midwest have been leaders in growing organic food-grade grains, and both have commercial mills as part of their operations to supply regional bakeries and consumers. The following lines were evaluated: 47.04 (‘Maxine’/’Gua’), 140.05 (‘Warthog’/’Gua’), 174.01 (‘Clarks Cream’/’Arapahoe’), 202.07 (‘Rouge de Bordeaux’/’Arapahoe’), 212.08 (‘Red Fife’/’Arapahoe’), 260.06 (‘Rouge de Bordeaux’/’Warthog’), and ‘Warthog’ was used as the commercial check on all farms. Lines to be evaluated at the farm were selected in a participatory manner after field-days and meetings with farmers, bakers, millers, and other stakeholders using the on-station data based on winter survival, grain yield, agronomic performance, resistance to FHB and other diseases evaluated on-station. On-farm experiments were planted in a RCBD with two replications in each location with a plot size of 18.5m^2^ (3 × 6.2 m) and 97.5 m^2^ (15 × 21.3 m) for 2020 and 2021. Planting was on September 26, 2019, and October 11, 2020, at Ridgeway, October 3, 2019, at Danforth, and October 21, 2019, at Freeville. Harvest date was July 16, 2020, and July 17, 2021, at Ridgeway, on July 7, 2020, at Danforth, and July 22, 2020, at Freeville. Experiments were managed by the participating farmers according to their standard practices for certified organic winter wheat at each farm, except that the Freeville location was managed by the Cornell University Small Grains Team. The Freeville trial was still included as part of the on-farm trials due to the nature of trials including plot size and number of breeding lines evaluated. Traits evaluated were grain yield and DON content. Grain yield was harvested with an experimental plot combine (Zurn 150 plot harvester) at Ridgeway and a Winterstiger plot master at Danforth. A bulked subsample of 100 g of grain from both replications was sent to the Wisconsin Crop Improvement association for DON content testing. Deoxynivalenol (DON) concentration in grain was obtained using an immunochromatographic assay for quantitative determination of DON (Accuscan Gold, Neogen Ltd).

#### On-farm genotypic statistical analysis

Empirical best linear unbiased estimates (BLUEs) for grain yield of each genotype for each location and year (i.e., environment) were estimated using plot-level information from all genotypes planted at a given environment and correcting for experimental design following the linear mixed model described in (1) but without using spatial corrections. A Dunnet test (*α* = 0.05) was used to compare the performance of the experimental lines to the ‘Warthog’ check.

### Participatory baking trials and sensory evaluations

Participatory baking trials and sensory evaluations were conducted in 2020 and 2021 to evaluate the performance of lines for artisan baking. Grain was obtained from large seed increase plots grown on-station in the experiments described above. Grain from Madison was used in 2020, while a proportional mix of grain from Madison and Freeville was used in 2021 (70% NY and 30% WI). Lines were selected based on on-station trial performance, DON levels, and grain availability. All grain was tested for DON concentration prior to milling to ensure food safety. Grain was also tested for protein content and falling number to better interpret the results of the baking trials. However, the values of protein content and falling number were not provided to bakers prior to the baking tests to avoid biasing expectations. We used the process described in Kucek et al. (2017) to evaluate the breeding lines and checks in naturally leavened (sourdough) bread. Briefly, this involves creating a levain out of the flour of each genotype, then using the same formula and process for each genotype to create a sourdough bread. Bakers score the mixing, proofing, and shaping stages of dough development, followed by scores for the exterior, interior and flavor of the final bread. Scores are given on a 10-point scale from poor to optimal, with notes to indicate why a variety may not have scored optimally since there can be multiple reasons for non-optimal performance.

#### 2020 Baking trial

Four breeding lines 140.05, 174.01, 212.08, 260.06, and ‘Warthog’ as a commercial check were milled by Madison Sourdough Bakery on an Osstiroller 700 MSM Combi Mill with 28-inch stones. The flour was slightly sifted to achieve approximately 95% extraction for each genotype. The sifting process removes bran flakes above a certain particle size, and this was kept constant across all genotypes. The breeding lines and check were evaluated in a baking test conducted on March 11 and 12, 2020. Six professional artisan bakers traveled to Madison for the evaluation and participated over the two days. The bakers who participated in the evaluation included Melina Kelson of Bootleg Batard in Chicago, IL, Solveig Tofte of Sun Street Breads in Minneapolis, MN, Greg Wade of Publican Quality Bread in Chicago, Kirk Smock of ORIGIN Breads in Madison, WI, Matt Kronschnabel of Bard Bread in Viroqua, WI, and Andrew Hutchison of Madison Sourdough. The six bakers collectively worked on each variety during the baking process and adjustments were made by consensus for each variety to optimize the level of hydration, rest time, and mix time without changing the base formula (other than hydration). Each baker then individually assigned scores to each variety during each step of the process.

#### 2021 Baking trial

Two breeding lines 47.04, 260.06, and ‘Warthog’ as commercial check were milled and evaluated in the remote baking tests conducted during the week of 5 May 2021. Remote tests were done in 2021 due to COVID-19 restrictions. The grain was milled by Meadowlark Organics in Ridgeway, Wisconsin, on a Meadows 30″ stone mill (Meadows Mills Inc., North Wilkesboro, NC). The flour was slightly sifted to achieve approximately 85% extraction to follow the commercial standard protocol for Meadowlark Organic. This was determined to be the best point of comparison by the participating bakers as they are familiar with working with Meadowlark’s flour. The baking evaluation also included Meadowlark’s current commercial bread flour (12.4% protein blend of ‘Warthog’ winter wheat and a spring wheat variety) as a second commercial flour check. Flour was then shipped to each baker and were evaluated in a baking test done remotely by five bakers in their own bakeries, all of whom participated in the 2020 bake test. The same protocol was used from the 2020 trials but done individually by each baker. Because of the remote trial reducing support for bakers during the process, data collection was reduced to one score for each of the major phases of the baking process rather than each individual step, with additional room for bakers to write descriptive evaluations of each step in lieu of more detailed scoring.

#### Sensory evaluation

Bread from the baking evaluation was used the following day in a hedonic sensory evaluation by members of the public and the research team in 2020 and 2021. Each bread was rated for appearance, texture, and flavor on a 1–5 scale, with 5 being the most preferred. The sensory evaluation in 2020 was done at Madison Sourdough on March 11 with 55 participants. The sensory evaluation in 2021 was done remotely by 40 participants with kits assembled including all samples from Madison Sourdough and ORIGIN Breads in Madison, WI, and then tasted by individuals off-site due to COVID-19 restrictions.

#### Statistical analysis

Empirical best linear unbiased estimates (BLUEs) of each baking and sensory trait for each genotype for each year (i.e., environment) were estimated using baker’s and taster’s scores information from all genotypes tested at a given year (i.e., environment) with the following linear mixed model:

6 $$\underline{{y_{ij} }} = \mu + G_{i} + \underline{{\beta_{j} }} + \underline{{\varepsilon_{ij} }}$$

where *y*_*ij*_ is the score, *µ* is the overall mean, *β*_*j*_ is either the effect of the *j*th baker or taster with *β*_*j*_ ~ *N*(0, *σ*^2^_*β*_), and *ε*_*ijkl*_ is the residual term with *ε*_*ijkl*_ ~ *N*(0, *σ*^2^_*ε*_), with the covariance among random effects equal to zero and *σ*^2^_*β*_, and *σ*^2^_*ε*_ the evaluator and residual error variances, respectively. When dough, bread and bake summaries were analyzed, *Υ*_*k*_, a new term was included as the effect of the kth baking stage with *Υ*_*k*_ ~ *N*(0, *σ*^2^_*Υ*_). The *ε*_*ijk*_ is the residual term with *ε*_*ijk*_ ~ *N*(0, *σ*^2^_*ε*_), with the covariance among random effects equal to zero and *σ*^2^_*β*_, and *σ*^2^_*Υ*_, and *σ*^2^_*ε*_ the evaluator, baking stage, and residual error variances, respectively.

#### Results

### Genotype-by-environment interaction (GEI) and variance components

**Fig. 3:**
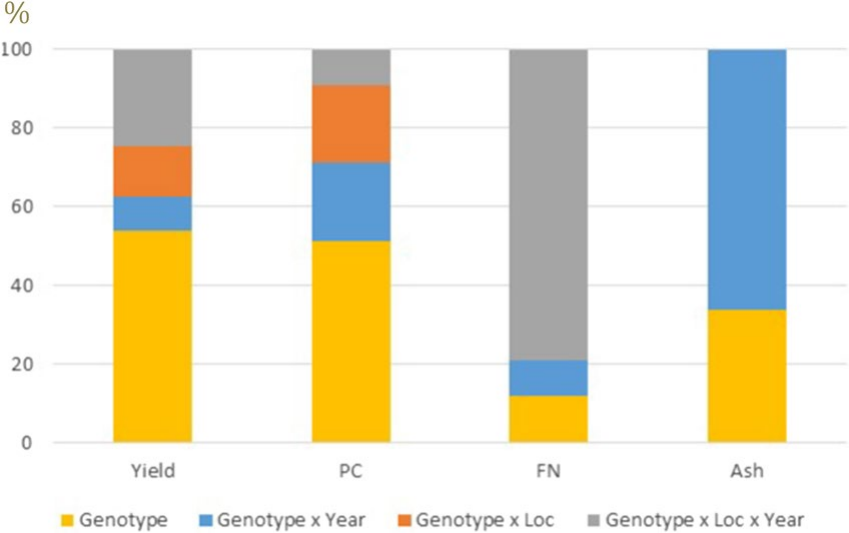
Relative proportion of all genetic variance components (G, GA, GL, GAL) for grain yield (Yield, 2017–2021), protein content (PC, 2019–2021), falling number (FN, 2019–2021) and ash content (Ash, 2020–2021). All the traits were evaluated in two locations (Madison and Danforth)

Approximately 54% of the total genetic variance (i.e., G + GL + GA + GLA) of grain yield was explained by the genotypic main effects (G) while the remaining 46% was explained by GEI (GL + GA + GLA), with genotype-by-location-by-year (GLA = 25%) being the largest effect (Fig. 3). Protein content also had a strong genotypic main effect, with 52% of the genetic variance component explained by genotypic main effects. On the other hand, GEI explained a large proportion of the total genetic variance of falling number and ash content (78% for falling number, and 66% for ash content; Fig. 3). Finally, although three mega-environments were identified in the GGE biplots (supplementary file 6), no repeatable pattern of GEI could be identified as drivers of those groups with mega-environments grouping environments across years and locations.

### Finlay–Wilkinson stability

**Fig. 4:**
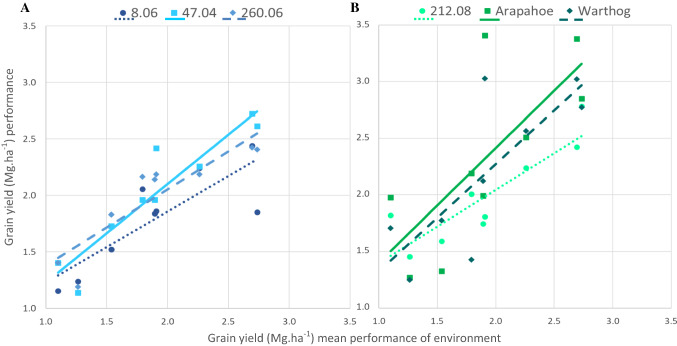
Finlay–Wilkinson regression for grain yield of hard red winter wheat breeding lines and commercial checks evaluated in seven environments in three locations (Madison, Danforth and Spooner) and five years (2017–2021). Only genotypes that showed the A) lowest (i.e., more stable, 8.06, 47.04, and *260.06*) and B) highest (i.e. less stable, *212.08,* ‘Arapahoe,’ and ‘Warthog’) values for the Wricke’s ecovalence stability coefficient (from Table 1, no extremes) are shown

Only small differences in FW slopes for grain yield were observed among genotypes, with regression slopes between 0.79 (212.08) and 1.20 (‘Warthog’) for all the environments (Table 1; Fig. 4). This indicates that similar dynamic stability exists among genotypes. On the other hand, R^2^ values were higher for most of the breeding lines than for the commercial checks, with values ranging from 0.91 (212.08) to 0.99 (47.04), and both checks, ‘Arapahoe’ and ‘Warthog,’ having values of 0.92, indicating higher predictability from the FW model for breeding lines than commercial checks (Table 1). Finally, because the years 2020 and 2021 in Madison were extremely high-yielding environments and regressions can be affected by extreme values, a regression model excluding the years 2020 and 2021 for Madison was evaluated. The range of the regression coefficients increased, while R^2^ values decreased after excluding the extreme values, including ‘Arapahoe’ and ‘Warthog’ that showed lower R^2^ values (0.19 and 0.30 for ‘Arapahoe’ and ‘Warthog’ respectively).

**Table 1:** Grain yield stability parameters of hard red winter wheat breeding lines and commercial checks evaluated in ten environments in two locations (Danforth and Madison) and five years (2017–2021)

Genotype	Overall stability				Stability (no extremes)		
	Mean	*β*_1_	*R*^2^	*W* (10^+6^)	*β*_1_	*R*^2^	*W* (10^+6^)
7.02	2766	1.06	0.98	0.86	1.00	0.85	0.40
8.06	2587	0.97	0.98	0.30	0.97	0.91	0.25
9.1	2750	1.07	0.98	0.98	0.76	0.55	0.76
11.03	2519	0.89	0.97	1.24	0.71	0.66	0.81
12.05	3082	1.11	0.95	1.56	0.93	0.41	0.95
47.04	2997	1.08	0.99	0.39	1.00	0.73	0.20
54.08	2655	0.95	0.98	0.71	0.58	0.37	0.63
138.04	2604	0.99	0.93	1.06	0.91	0.44	0.76
140.05	2689	1.00	0.96	0.97	1.00	0.43	0.66
145.06	2642	1.03	0.99	0.34	1.04	0.99	0.11
174.01	2769	0.97	0.96	0.93	1.30	0.68	0.47
183.05	2698	0.93	0.95	0.87	1.20	0.75	0.68
199.09	2750	0.93	0.96	1.27	0.93	0.80	0.93
202.07	2836	0.96	0.95	0.73	0.92	0.93	0.30
212.08	2639	0.79	0.91	2.17	0.41	0.34	0.68
260.06	2863	1.01	0.99	0.26	0.94	0.77	0.16
‘Arapahoe’	3206	1.07	0.92	1.87	0.97	0.19	0.94
‘Warthog’	3234	1.20	0.92	3.10	1.05	0.30	1.05

Finlay–Wilkinson regression coefficients (*β*_1_, values closer to zero indicate static stability while closer to one indicate dynamic stability) and nonlinear stability (*R*^2^ of FW, values close to 1 show more predictable performance), as well as Wricke’s ecovalence stability coefficient (*W*, lower values indicate dynamic stability) are shown. Because the years 2020 and 2021 were extremely high-yielding years in Madison (see supplemental file 1), values are also shown for the stability analysis without the years 2020 and 2021 in Madison (stability no extremes). Mean grain yield performance is shown for comparison purposes

### Wricke’s ecovalence stability coefficient

There were orders of magnitude of differences in Wricke’s ecovalence stability coefficients (W) for grain yield among genotypes, with W values between 0.26 × 10^6^ (260.06) and 3.10 × 10^6^ (‘Warthog’) (Table 1). A similar rank-order of genotypes was found when the extremely high-yielding environments were removed from the analysis (Table 1). The performance of genotypes with the highest and lowest W coefficients is shown in Fig. 4 for all the environments (excluding the extremes) to illustrate predictability across environments.

Mean protein content across years and locations was 10.7, in general, higher for the breeding lines than for the commercial checks ‘Arapahoe’ (10.5%) and ‘Warthog’ (10.1%, Table 2). Some breeding lines 260.06 (10.1%) and 47.05 (10.4%) were similar to the ‘Arapahoe’ and ‘Warthog’. Breeding lines 11.03 (11.5%) and 140.05 (11.9%) had significantly higher protein than ‘Arapahoe’ and ‘Warthog’ but were the least stable genotypes for protein content with *W* values of 1.92 and 2.23, respectively (Table 2). On the other hand, some of the breeding lines such as 8.06 have high protein (11.0%) contents with low values of *W* (*W* = 0.58). Falling number was between 300 and 400 s for all the breeding lines with a range of values for *W* (Table 2, supplementary file 4). Both ‘Arapahoe’ and ‘Warthog’ were among the most volatile genotypes for falling number, with *W* values of 12,431 and 13,183, respectively. Ash content was similar between breeding lines ranging between 1.64 and 1.88% and commercial checks ‘Arapahoe’ (1.79%) and ‘Warthog’ (1.90%) with small overall variance (Table 2, supplementary file 2).

**Table 2:** Mean and stability parameters of hard red winter wheat breeding lines and commercial checks evaluated in two locations (i.e., Danforth and Madison) and three years (i.e., 2019–2021) for protein concentration (PC), falling number (FN), and ash content (Ash)

Genotype	PC (%)	*W*_PC_	FN (s)	*W*_FN_	Ash (%)	*W*_Ash_ 10^+2^
7.02	10.4	1.95	322	1026	1.70	0.26
8.06	11.0	0.58	335	1699	1.66	2.50
9.1	10.7	0.30	317	2889	1.71	3.48
11.03	11.4	1.92	346	485	1.64	0.43
12.05	11.0	1.14	333	6386	1.69	0.26
47.04	10.4	0.86	323	3694	1.71	0.92
54.08	10.7	0.13	303	2254	1.72	2.02
138.04	10.7	0.13	342	4017	1.84	1.06
140.05	11.9	2.23	345	4762	1.88	1.52
145.06	10.7	2.00	361	7336	1.77	0.14
174.01	10.7	0.61	356	1435	1.66	0.87
183.05	10.6	0.44	344	6175	1.87	3.30
199.09	10.6	1.52	346	11,706	1.73	0.71
202.07	10.0	0.33	334	842	1.73	0.10
212.08	11.0	0.84	328	4204	1.77	0.24
260.06	10.1	0.20	332	6181	1.79	1.14
‘Arapahoe’	10.5	3.01	336	13,183	1.79	4.33
‘Warthog’	10.1	0.73	382	12,431	1.90	1.04

Wricke’s ecovalence stability coefficient (*W*) are shown for each trait. Ash content was evaluated in 2 years (2020–2021) in two locations (Danforth and Madison)

#### Participatory on-farm evaluations

There was a change in genotype ranking between on-station and on-farm for grain yield, even within the same year (Table 3). The on-station experiments showed that the selected breeding lines were not different from ‘Warthog’ in most of the environments (supplementary file 1, Table 3). But the breeding lines 140.05 (4525 kg ha^−1^), 47.04 (6074 kg ha^−1^), and 260.06 (5196 kg ha^−1^) were superior in yield to the commercial check ‘Warthog’ (2711 kg ha^−1^) in Ridgeway in 2020. Breeding lines 212.08 (2783 kg ha^−1^) and 140.05 (4525 kg ha^−1^) were inferior to ‘Warthog’ (3824 kg ha^−1^) in Ridgeway in 2021, and no differences were found among genotypes in Danforth, 2020 (Table 3). Breeding line 260.06 was not different from ‘Warthog’ in Freeville 2020, while breeding lines 47.04 and 174.01 were significantly inferior to ‘Warthog’. All the breeding lines had DON concentration levels under 1 ppm except breeding line 140.05 in Ridgeway 2020 and 140.05 and 212.08 in Ridgeway 2021 (supplementary file 7).

**Table 3:** Grain yield (kg ha^−1^) performance of breeding lines and commercial checks in on-farm trials during the 2020 and 2021 years, and summary information for the same years of on-station performance

Genotype	Wisconsin						Illinois				New York	Overall
	On-station				On-farm		On-station			On-farm	On-farm	
	Madison			Spooner	Ridgeway		Danforth			Danforth	Freeville	
	2020	2021	2017–2021	2021	2020	2021	2020	2021	2017–2021	2020	2020	2017–2021
212.08	5054	4555^B^	3429	1618	–	2783^B^	1795	2781	2001	–	–	2639
140.05	4473	5994^B^	3653	1597	4525^A^	3293^B^	1838	2261	1940	1425	–	2689
174.01	4991	5216^B^	3715	1309	2157	–	1964	2387	2033	1484	3706^B^	2769
202.07	5223	5176^B^	3652	1467	–	3439^0^	2420	2647	2219	–	–	2836
47.04	5123	6235^B^	3917	1759	6074^A^	3516^0^	2017	2614	2225	1642	3807^B^	2997
260.06	5059	5920^B^	3762	1861	5196^A^	3713^0^	1795	2406	2093	1805	4195^0^	2863
‘Warthog’	4865	7443^0^	4387	1805	2711^0^	3824^0^	2238	2773	2193	1805	5260^0^	3234
S.E	471	463	449	232	195	175	253	295	324	175	563	434
Dunnet	1115	1096	1064	549	461	414	600	699	768	414	1333	1028

Genotypes were compared to the check ‘Warthog’ with a Dunnet test within trial. A represents mean performance superior to the check, B represents mean performance inferior to the check, all other mean performances are not different from the check performance by the Dunnet test at a 5% level of significance

#### Baking test and sensory evaluations

**Fig. 5:**
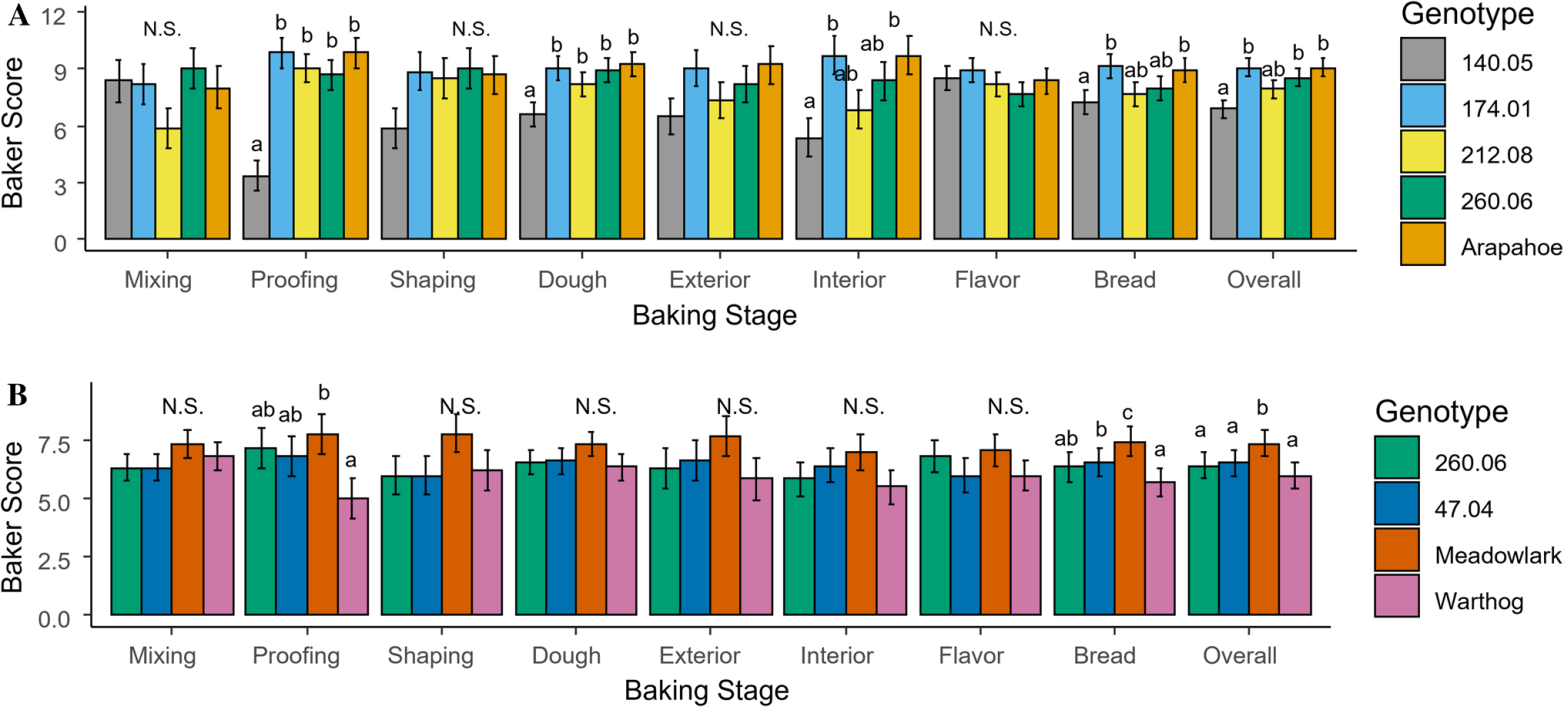
Baking trial test of hard red winter wheat breeding lines and commercial checks evaluated in naturally leavened artisan bread trials in 2020 and 2021. **a** 2020 Madison Sourdough baking test. **b** 2021 Remote baking test. Data is presented as the component traits (mixing, proofing shaping, exterior, interior, flavor) followed by the composite traits (dough, bread) for the pre- and post-bake phases, along with an ‘overall’ score for the whole baking test

All the breeding lines evaluated in the 2020 bake tests had suitable baking properties compared to the commercial check (Fig. 5). The breeding line 140.05 was unstable in the short fermentation test with an overall poor performance (Fig. 5) but performed better in the long-fermentation test and some bakers identified it as their favorite breeding line in the long-fermentation evaluation (data not shown). The breeding line 174.01 was stable and the best performing genotype at most stages during the baking process (Fig. 5). The bakers described the breeding line 212.08 as insufficient for extensibility during mixing compared to other lines but was close to optimal for the rest of the process (Fig. 5). Breeding line 260.06 was close to the check throughout the baking process (Fig. 5). The check, Arapahoe, was rated slightly insufficient for extensibility by the bakers during mixing and for proofing strength, and then close to optimal for the rest of the process (Fig. 5). All breeding lines were rated similarly to the check for flavor which is promising for commercialization. In the 2021 bake tests, the commercial Meadowlark bread flour (a blend including high-protein spring wheat) performed the best while the commercial check ‘Warthog’ performed the worst (Fig. 5). The two breeding lines had suitable baking properties for high-quality artisanal bread at lower protein concentrations than the Meadowlark bread flour. The breeding line 260.06 was stable throughout the baking process, similar to the Meadowlark bread flour, and better than ‘Warthog’. This line was present in both years and performed well compared to other breeding lines and the checks. Although some breeding lines were different from the commercial checks at some stages in the baking trials, there were no statistical differences between breeding lines and checks for sensory traits in either year (data not shown).

## Discussion

### Breeding strategies and characterization of selection

The breeding lines advanced and selected in our program have better grain quality than the checks and appear more stable for grain quality and grain yield without compromising grain yield. Even though this project started and had its initial stages in the US Northeast, the genetic diversity available was still relevant in the US Upper Midwest probably because of similar breeding goals. There are currently no cultivars of hard winter wheat developed in the US Upper Midwest available to farmers, and until this work started in 2012, there were no breeding programs with the explicit goal of breeding and selecting under organic conditions in the US Upper Midwest. Furthermore, these plant breeding efforts are unique in the USA by targeting high-end artisan baking quality, although programs with similar goals and methods exist in Europe (Löschenberger et al. 2008; Wolfe et al. 2008; Dawson et al. 2011; Osman et al. 2012) and in the USA for spring wheat (Hills 2012; Kucek et al. 2017). Seven cycles of selection under certified organic conditions including two cycles of selection for FHB and protein in the Northeast (New York) and five cycles of selection for adaptation in the US Upper Midwest (Illinois and Wisconsin, Fig. 2) resulted in a set of 16 high performing breeding lines that perform well in terms of agronomic, disease, and grain quality performance. Strong selection pressure was used initially in the breeding program to discard 98% of the lines based on tolerance to FHB from an FHB inoculated nursery and a tandem selection for protein content. Later, the largest driver of selection was adaptation to the US Upper Midwest and disease resistance. We discarded eight breeding lines in 2017 that did not survive the winter in either location. Additionally, one line was discarded because it was the only breeding line showing a severe infection of glume blotch (*Phaeosphaeria nodorum*) which can severely reduce the quality of the grain. In 2018, we discarded seven breeding lines with a high level of natural infection to FHB, eight lines with low grain yield in either location, and four lines due to poor winter survival in either location in combination with a low grain yield in 2017 or 2018. A strong selection for grain yield performance occurred in 2019 where overall performance across six environments in three years was used to discard eight lines based on general poor or inconsistent performance. Stability for grain yield was not explicitly estimated until the fourth year where genotypes had sufficiently sampled a range of environmental conditions. The core population of selected breeding lines therefore consisted of 16 F_3:8_ breeding lines representing 64% of the F_2:3_ families. The 16 breeding lines represent nine of the original crosses combining eight parents. The core breeding population has a high level of genetic diversity (supplementary files 1–5) and has the potential for at least one commercial release as well as continuing additional breeding cycles to combine complementary traits.

Our experimental lines are F3-derived populations and therefore retain a higher level of genotypic and phenotypic diversity. The practice of deriving populations early in the breeding program is not un-common in the development and release of cultivars and has been implemented by others (Jones et al. 2006; Haley et al. 2007, 2014). In our case, the lines maintain sufficient plant height, maturity, and overall phenotypic uniformity to meet cultivar certification standards. As part of our interactions with farmers involved in on-farm trials and field visits, farmers encouraged us to maintain this level of variability within a cultivar as no harvest or any other logistical challenges were expected because the diversity for critical management traits such as plant maturity and plant height was small. This practice is also common in the context of evolutionary breeding, where Bocci et al. (2020) report similar preferences by farmers to higher diversity. Some of the reported advantages of retaining higher levels of genetic diversity are associated to higher resilience and stability (Bocci et al., 2020). Additionally, remnant genetic diversity could be exploited up to a certain extent by farmers that save seeds by providing further local adaptation (Bocci et al. 2020; Wolfe & Ceccarelli 2020). In our context, an excessive level of phenotypic diversity could pose additional challenges when preparing breeder’s seed. Therefore, special care to rogueing off-types needs to be applied in the development of breeder’s seed to maintain phenotypic uniformity for cultivar release (i.e., Haley et al. 2007). The International Union for the Protection of New Plant Varieties (UPOV) recommends two strategies for evaluating and maintaining uniformity in new varieties: family structure with mild rogueing (i.e., using 100 head-to-row with a maximum of 3% of off-types), or large populations with strong rogueing (i.e., using 2000 plants with a 0.3% of off-types) (UPOV 2017). Haley et al. (2007) suggests using several generations of planting and off-type rogueing as a method to maintain diversity while meeting UPOV standards. We believe that a modification of the family structure method could provide the same results with a shorter time to release. This would require a larger number of head-to-row families (i.e., maybe 400–500), heavy rogueing of off-types (1%), and bulking into a dynamic mixture (Wolfe and Ceccarelli 2020) while maintaining a head from each head-row for the next cycle of breeder seed.

### Trait performance and GEI

#### Disease resistance

FHB can be caused by several species of the *Fusarium* and *Microdochium* genera, but is dominated by *Fusarium graminearum* Schwabe in the US Upper Midwest (Gale et al. 2007; Vaughan et al. 2020). It is one of the most damaging diseases in wheat because it has a direct effect on grain yield, and because grain contaminated with deoxynivalenol (DON) is a health hazard for people and animals (Su et al. 2019). Therefore, grain with high levels of DON cannot be sold to food markets and needs to be sold at a lower price (Su et al. 2019). The development of FHB is favored by wet weather during flowering, which often occurs in the US Upper Midwest and Eastern USA. We had a strong selection for FHB using inoculated nurseries early in our breeding cycle and natural infection later. The commercial check ‘Warthog’ did not meet food-grade levels during our first year of baking trials and could therefore not be used for baking. Some of our selected breeding lines were able to meet food safety parameters consistently. Our work in collaboration with multi-year national efforts to breed cereal grain varieties with higher tolerance to FHB including the US wheat and barley scab initiative will be of great benefit to both organic and conventional breeding programs (USWBSI 2019).

#### Agronomics and grain yield

Although GEI was relevant in our study, over 50% of the total genetic variance was due to genotypic main effect for grain yield and protein content. The remaining GEI did not represent repeatable GEI effects such as genotype by location or management practices and is therefore more difficult to predict. The strong effect of the genotype-by-location and genotype-by-location by year interactions for grain yield is commonly found in other species (Gutierrez et al. 2015; Monteverde et al. 2018, 2019; González-Barrios et al. 2019; Bhatta et al. 2020; Neyhart et al. 2021a,b) and is concordant with wheat results (Lado et al. 2016; Kucek et al. 2019), with the difference that mega-environments do not show repeatable sources of GEI in our study (supplementary file 6). Our study included two locations evaluated during five years, with only a third location incorporated during the last year. A small genotype-by-location variance could be the result of fewer locations being tested but could also mean that organic management systems in the US Upper Midwest may represent a coherent target population of environments, within one mega-environment, with existing GEI due largely to unrepeatable causes.

Organic farmers identified weed competitive ability as a trait of interest (Kucek 2017), likely due to limited weed control methods in organic systems. Genotypes with good weed competitive ability often have higher plant biomass and moderate to high plant height (Kissing Kucek et al. 2021). We found that the breeding lines that advanced to the later stages of evaluation are tall or have intermediate to high plant height. Although we did not evaluate weed competitive ability directly in our study, we believe that weed competitive ability might be an underlying reason for the superior performance of taller genotypes in our program. In some cases, tall plants might exhibit higher lodging incidence; however, our tall breeding lines exhibited a low incidence of lodging that might have contributed to their advantage.

#### Baking quality

Protein content is one of the most frequently used proxy traits for baking quality even though it does not fully explain baking quality properties (Borghi 1999; Gabriel et al. 2017). Baking quality is measured as the volume of bread by unit of flour. The relationship between baking quality and protein is not linear as quality increases with protein until 12%, and it might decrease with higher values of protein (Timms et al. 1981; Gabriel et al. 2017). This relationship is dependent on the baking method (Færgestad et al. 2000; Tronsmo et al. 2003). Therefore, protein content standards developed for industrial baking (Færgestad et al. 2000; Tronsmo et al. 2003) might be completely disconnected from requirements for artisanal baking where longer fermentation methods might be used (Ross 2018). The ratios between protein components are as important for baking quality as the total amount of protein (He et al. 2005). The protein quality is often more strongly related to genotype than overall protein content. For example, in French seed markets, varieties obtain an overall quality rating when released, which is independent of their protein content in any particular year. Breeding for stable protein levels and evaluating breeding lines for baking quality directly can result in better recommendations for farmers about which varieties have consistently good quality.

There is a long tradition of grain quality standards for bread wheat quality under conventional production that meet the standards of industrial milling and baking (Hills 2012; Sanchez-Garcia et al. 2015), but no such standards have been developed for artisanal baking. Flour quality is affected by a large number of factors including wheat category, milling process (Gómez et al. 2020) including particle size (Ross and Kongraksawech 2018; Alava Vargas 2020; Gómez et al. 2020), level of extraction (Baasandorj et al. 2020), and even environmental conditions (Caffe-Treml et al. 2011). In any case, for the most part, standards developed for flour quality are good predictors of industrial bread quality for refined breads (Khalid et al. 2021). However, it has been difficult to establish the relationship, if any, between the laboratory standards developed for industrial baking with refined flour to the quality of the artisanal baked breads (Ross 2018; Krill-Brown et al. 2019).

There are three main differences between industrial and artisanal baking: adaptability, mixing, and the use of sourdoughs. The human touch and the possibility of corrections during artisanal baking allows the baker more flexibility in adapting to the optimal conditions for each specific flour (Ross 2018). The amount and methodology used for mixing in artisanal baking is quite different compared to industrial baking (Ross 2018; Krill-Brown et al. 2019). The use of a sourdough instead of yeast, has a significant impact in the rheological characteristics of the dough (Angioloni et al. 2006) and on the aromatic compounds (Xi et al. 2020). Protein content and protein quality are still relevant traits for baking quality in artisanal baking (Hills 2012; Kucek et al. 2017), but quality parameters for artisan baked whole grain bread are not the same as for industrial bread baking that is based on ultra-refined flour (Ross 2018). Due to the longer sourdough fermentation and typically less intensive mixing process used by artisanal bakers, genotypes that do not meet the 12.5% of protein content required by industrial milling may still perform well in artisanal processes. This suggests that it may be possible to develop varieties of winter wheat that perform well in artisanal baking at lower protein concentrations if they have good protein quality. Furthermore, Ross (2018), working with whole bread sourdoughs found positive correlations between the loaf characteristics and some laboratory dough strength evaluations, and indicated that the results from the extensograph maximum resistance evaluated at 135 min (Rmax135) could be used for evaluating quality in long-fermentation doughs. Other alveograph values (i.e., W and P) have been useful in artisanal bread evaluations for example for French breads (Ross 2018; Calvel et al. 2001). In summary, different levels of product development input will be needed to develop standards for artisanal bread products, including baker testing and consumer tasting. Although more challenging to implement, these evaluations will facilitate the incorporation of a broader set of users in the decision making process of end products for variety development (Dawson et al. 2011). High grain quality and flavor for artisanal baking are especially important traits in organic agriculture (Hills et al. 2013).

A strong genotypic main effect for protein content was observed in our study and has also been observed in other studies (Williams et al. 2008). We observed that diversity is still present for protein content among our breeding lines. These can be explained by the strong selection intensity for protein content applied in early generations, while no further selection for protein content was done at later stages of selection. Based on the artisanal baking tests, even though breeding lines were tested at lower than typical protein contents for industrial processes, they performed well when compared to commercial checks. Further studies need to be developed to explain the relation between artisanal baking and protein content. Falling number is a complex trait that depends on several factors and has a strong environmental influence (Johansson 2002; Kucek et al. 2017; Sjoberg et al. 2020). Due to the high environmental effects found in our study, to have a better evaluation of the relationship between falling number mean values and the stability, a larger dataset would be required. Ash content showed that two thirds of the genetic variation was explained by the genotype-by-year interaction, while the remaining third was explained by genotypic effects. These high levels of GEI are similar to results reported in other studies (Morris et al. 2009; Ficco et al. 2020). Even though one third of the genetic variation is associated with genotypic main effects, a very small range of values was observed in our study. Grain size was correlated with ash content in some studies, including a durum wheat study (Ficco et al. 2020). This would be expected because the ash measures the bran-endosperm ratio. As an incidental observation, breeding lines with the lowest ash content seem to have plumper grain (i.e., 11.03), unfortunately, we did not measure size and shape of the grain in this study.

### On-farm trials

We found crossover GEI between on-farm and on-station performance for grain yield. This difference in performance between research stations and on-farm trials has been documented in the literature (Simmonds 1980, 1991), and given the prevalence of genotype-by-location interactions in multi-environment trials, this is not remarkable (Annicchiarico 2002; Annicchiarico et al. 2006). On the other hand, the variation among on-station trials was higher than the differences between farms/years and research stations. Another important consideration in the interpretation of the on-farm results is that they are based on two years of data in two locations, and given that genotype-by-year and genotype-by-year-by-location interactions were substantial in our study, and are commonly reported as relevant in other studies (Kucek et al. 2019), caution should be exercised with the interpretation of these results.

### Baking test

Our baking tests represent an exploratory approach to incorporate bakers in the process of choosing promising lines. Because of the large amount of grain needed for a baking trial and the small number of lines that can be tested at once, we were not able to test all lines in both years. The baking tests are time intensive and logistically difficult but worth the effort to obtain feedback from professional bakers prior to release of varieties specifically targeted at that market. The results from the baking trials, even though preliminary, are promising, and we will continue to test these lines prior to release. The results in the baking trials do not necessarily match the laboratory baking quality evaluations (supplementary file 5) and reports in the literature (Timms et al. 1981; Færgestad et al. 2000; Tronsmo et al. 2003; Sanchez-Garcia et al. 2015; Gabriel et al. 2017). Further work should identify or develop better laboratory tests that will allow selection of lines in early stages and strategic use of baking tests in the final stages on the process. The development of these laboratory resources for conventional programs aided selection for quality traits needed by industrial bakers. Reliable tests for artisanal baking quality that can be conducted with small grain samples will help in selection for the quality parameters needed by this market.

### Breeding for grain yield stability

All breeding lines and the checks showed a dynamic stability for grain yield with different levels of performance of the FW linear model. The checks used in our experiments, ‘Warthog’ and ‘Arapahoe,’ were less stable than all of the breeding lines, showing a larger lack of fit of the FW regression (i.e., smaller *R*^2^) and larger values for the Wricke’s ecovalence stability coefficient, indicating a more volatile response to the environment. Furthermore, some of the breeding lines combine high grain yield performance similar to the checks with more stable performance and a dynamic response to the environment. This combination of agronomic performance with stability is valuable in general, but especially under organic production systems where more diverse management practices are used (Wolfe et al. 2008; Lammerts van Bueren et al. 2011). A possible explanation for the high stability and performance could be the level of remnant genetic diversity present in some of these lines. The breeding lines were F3-derived, when many loci were still segregating, maintaining some level of genetic diversity within the lines. In winter wheat populations with very high levels of genetic diversity (mixtures or composite crosses), a higher level of phenotypic plasticity has been reported (Weedon and Finckh 2019). The advantages of retaining more genetic diversity in breeding populations and farm fields have been extensively documented in cereals (Allard 1961; Wolfe and Ceccarelli 2020; Bocci et al. 2020). While our breeding lines are not as genetically diverse as the populations studied by those authors, there is some remaining phenotypic and genetic variability that may contribute to their stability.

Another possible explanation for the stable performance of these lines might be associated with the Jinks–Connolly rule (Jinks and Connolly 1973) and selection in multiple environments. The breeding implications of GxE were first proposed by Wright (1939), Haldane (1946), and later Falconer and Latyszewski (1952) who shifted the focus of GxE to a correlated characters problem. Hammond (1947) proposed that selection should be conducted on the most favorable environment (Hammond’s conjecture) because the best environment would discriminate genotypes better with higher heritability and less environmental variance. This theory was supported by multiple breeders (Frey 1964; Roy and Murty 1970; Fasoulas 1973) and opposed by several authors who argued in favor of selection in the targeted environments (Wright 1939; Lush 1945; Nichols 1947; Kelley 1949). Selection conducted only on the best environments might favor genotypes that are more sensitive to the environment, decreasing their stability. Jinks and Connolly (1973) suggested that an antagonistic selection where the breeder may up-select in a bad environment and down-select in a good environment could reduce environmental sensitivity (and improve stability) and other authors provided experimental evidence supporting this theory (Falconer 1990). Therefore, early selection of lines in multiple and contrasting environments could have favored less-sensitive lines with overall good performance. Walsh and Lynch (2018) propose to go further and use an index selection to select for overall performance and sensitivity.

Our breeding lines have been selected during seven cycles under organic management practices, while the checks were selected under conventional management. Therefore, our breeding lines might also be more adapted to the specific environments of organic production. While selection in the target environment is key to achieving the best response to selection, this principle has not always been applied for breeding for organic production as this often represents a small market for conventional breeding programs. As a result, many lines used by organic farmers were developed under conventional management and then tested in organic systems. Unsurprisingly, studies have documented better adaptation to organic management when selecting under organic conditions (Reid et al. 2009; Bocci et al. 2020). Because of the interrelated nature of organic, local, and artisanal grain markets as a growing high-value option for farmers, we have developed a breeding program focused on this target environment and market, without a parallel conventional program. While the program is smaller than many conventional programs, it is focused on regional adaptation to organic systems and artisanal food end-uses, which allows us to prioritize traits that may be unique to organic systems such as stable production and quality. Stability is important in all systems, but may be more relevant in organic production systems than in conventional systems (Knapp and van der Heijden 2018). It is therefore necessary to take stability into consideration to select varieties that can perform well across the diverse landscape of management systems that goes under the umbrella of organic production. There are multiple definitions of stability in plant breeding, and therefore, the interpretation of stability results should be performed carefully. We tried to focus on both spatial (i.e., location) as well as temporal (i.e., years) stability in our study, and we used both dynamic and static stability indicators. In this context, static stability refers to genotypes that perform similarly across environmental conditions (Becker and Leon 1988), while dynamic stability refers to genotypes that maintain a relative performance according to the environmental mean and therefore respond to changes in environmental conditions (Finlay and Wilkinson 1963). Dynamic stability is usually sought for traits such as grain yield while static stability is usually desirable for quality traits (Becker and Leon 1988), where a predictable and consistent product is advantageous for the industry and to sustain long-term profitability of regional grain systems.

## Conclusions

The results presented here show that a small-scale breeding program with a distinct focus can produce relevant lines for an important emerging market. We have used the results of these trials to continue the process of crossing and selection to build a breeding pipeline that can continue to deliver improved germplasm for farmers in the US Upper Midwest. While our lines have similar average performance to existing checks, the average performance does not capture the critical aspect of stability for farmers and bakers in regional grain systems. By choosing parents based on both high artisanal bread making quality and reliable performance in organic trials, we have been able to select for improved stability of performance and quality. The fact that we can select more stable lines than existing commercial checks for both production and quality traits at the beginning phases of a breeding program is a promising indicator of the potential to develop high performing, stable varieties for the US Upper Midwest. These results suggest that stable lines can be developed using a participatory breeding approach under organic management. Crop improvement explicitly targeting sustainable agriculture practices for selection with farm to table participatory perspectives are critical to achieve long-term sustainable crop production.

## Supplementary Information

Below is the link to the electronic supplementary material.

Supplementary file1 (DOCX 706 kb)

## Data Availability

All data will be deposited in the T3 database (https://wheat.triticeaetoolbox.org/). Researchers interested in plant materials should contact corresponding authors.

## Abbreviations

BLUEs: Best linear unbiased estimates
BYDV: Barley yellow dwarf virus
DON: Deoxynivalenol
FHB: Fusarium head blight
FW: Finlay-Wilkinson regression
GEI: Genotype-by-environment interaction
RCBD: Randomized complete block design
W: Wricke’s ecovalence stability coefficient

## Code availability

Code is available as supplementary material.
