# Supplementary material for: Developing high-quality value-added cereals for organic systems in the US Upper Midwest: hard red winter wheat (Triticum aestivum L.) breeding
Source: Theor Appl Genet. 2022 May 28;135(11):4005–27. doi: 10.1007/s00122-022-04112-0 (PMC9142347; doi:10.1007/s00122-022-04112-0)
Supplement: Supplementary file 1 — Supplementary file1 (DOCX 706 kb) [file 122_2022_4112_MOESM1_ESM.docx]

**Supplementary file 1.** Best linear unbiased estimates (BLUEs) of grain yield (kg ha^-1^) of hard red winter wheat breeding lines and commercial checks evaluated in eleven environments in three locations (Madison, Danforth and Spooner) and five years (i.e. 2017-2021). Trial mean, standard error (S.E.), Fisher’s least square differences (LSD) for α=0.05, heritability (H^2^), and whether a post-blocking row and/or column effect was used to obtain BLUEs is reported.

|  | Madison | | | | | | Spooner | | | Danforth | | | | | |  |
| --- | --- | --- | --- | --- | --- | --- | --- | --- | --- | --- | --- | --- | --- | --- | --- | --- |
| **Genotype** | 2017 | 2018 | 2019 | 2020 | 2021 | 5-yr |  | 2021 |  | 2017 | 2018 | 2019 | 2020 | 2021 | 5-yr | Overall |
| **7.02** | 2836 | 1870 | 4736 | 4763 | 6209 | 3776 |  | 1517 |  | 1678 | 1995* | 1182 | -- | 2118 | 1964 | 2766 |
| **8.06** | 2399 | 1859 | 4368 | 4875 | 5497 | 3612 |  | 1521 |  | 2056 | 1838 | 1244* | 1805 | 1852 | 1810 | 2587 |
| **9.1** | 2398 | 1964 | 3397 | 5377* | 5761 | 3610 |  | 1504 |  | 2166* | 1879 | 1209 | 1658 | 2403 | 2089* | 2750 |
| **11.03** | 2453 | 2076 | 4358 | 4842 | 4832 | 3376 |  | 1389 |  | 1662 | 1858 | 1208 | 1552 | 1961 | 1807 | 2518 |
| **12.05** | 3643 | 2907* | 5530* | 5597* | 6318 | 3938 |  | 1414 |  | 1877 | 1885 | 1421* | 1552 | 2333 | 1996 | 3082* |
| **47.04** | 2920 | 2417 | 4495 | 5123 | 6235 | 3917 |  | 1730* |  | 1960 | 1961* | 1142 | 2017 | 2614* | 2225* | 2997* |
| **54.08** | 3041 | 2348 | 5053* | 4773 | 5545 | 3460 |  | 1528 |  | 1633 | 1800 | 1343* | 2133 | 2218 | 2024 | 2655 |
| **138.04** | 2306 | 1103 | 4824 | 4460 | 5744 | 3285 |  | 1514 |  | 2056 | 1734 | 1062 | 2217* | 2152 | 2081* | 2604 |
| **140.05** | 2195 | 2725* | 3721 | 4473 | 5994 | 3653 |  | 1571 |  | 1517 | 1748 | -- | -- | 2261 | 1940 | 2689 |
| **145.06** | 2362 | 1898 | 3997 | 5052 | 5731 | 3658 |  | 1581 |  | 1784 | 1891 | 1250* | -- | 2181 | 1844 | 2642 |
| **174.01** | 2117 | 2650 | 4364 | 4991 | 5216 | 3715 |  | 1287 |  | 2380* | 1746 | 1375* | 1964 | 2387 | 2033 | 2769 |
| **183.05** | 1833 | 1610 | 3493 | 4741 | 5359 | 3497 |  | 1724 |  | 2265* | 2051* | 1251* | 1784 | 2216 | 2101* | 2698 |
| **199.09** | 2153 | 1548 | 4378 | 5074 | 5245 | 3515 |  | 1275 |  | 1962 | 1790 | 1342* | 1784 | 2751* | 2130* | 2750 |
| **202.07** | 2626 | 1759 | 4561 | 5223 | 5176 | 3652 |  | 1443 |  | 1863 | 1898 | 1233 | 2281* | 2647* | 2219* | 2836* |
| **212.08** | 3793 | 1808 | 4460 | 5054 | 4555 | 3429 |  | 1591 |  | 2008 | 1747 | 1463* | 1795 | 2781* | 2001 | 2639 |
| **260.06** | 2922 | 2185 | 4515 | 5059 | 5920 | 3762 |  | 1830* |  | 2165* | 2140* | 1195 | 1795 | 2406 | 2093* | 2863* |
| ‘Arapahoe’ | 4119 | 3408* | 4867 | 4871 | 6699 | 4159* |  | 1328 |  | 2191* | 1991* | 1276* | 2502* | 2850* | 2355* | 3206* |
| ‘Warthog’ | 3557 | 3028* | 4221 | 4865 | 7443 | 4387* |  | 1775* |  | 1430 | 2124* | 1256* | 2238* | 2773* | 2193* | 3234* |
| **Trial mean** | 2293 | 2155 | 4357 | 5001 | 5748 | 3719 |  | 1529 |  | 1793 | 1886 | 1255 | 1958 | 2383 | 2050 | 2793 |
| **Trial S.E.** | 35.5 | 80.0 | 52.4 | 70.8 | 44.0 | 18.2 |  | 14.0 |  | 35.5 | 24.3 | 31.6 | 45.0 | 23.3 | 13.3 | 12.5 |
| **LSD** | 363.5 | 733.9 | 453.6 | 530.4 | 439.6 | 426.0 |  | 100.4 |  | 224.1 | 225.2 | 236.7 | 331.4 | 280.0 | 302.0 | 410.4 |
| **Row** | Yes | Yes | No | No | Yes | Yes |  | Yes |  | No | No | No | No | Yes | No | No |
| **Col** | No | No | Yes | No | No | Yes |  | No |  | No | No | No | No | Yes | No | No |
| **H^2^** | 0.73 | 0.65 | 0.84 | 0.44 | 0.94 | 0.76 |  | 0.86 |  | 0.92 | 0.75 | 0.37 | 0.85 | 0.81 | 0.53 | 0.80 |

Genotypes that are not significantly different (α=0.05) from the highest yielding genotype in the trial are marked with a star. Danforth 5-year mean, and overall mean did not show significant differences among genotypes for grain yield.

**Supplementary file 2.** Best linear unbiased estimates (BLUEs) of test weight (g 500 ml^-1^) of hard red winter wheat breeding lines and commercial checks evaluated in seven environments in two locations (Madison and Danforth) and four years (i.e. 2018-2021). Trial mean, standard error (S.E.), heritability (H^2^), Fisher’s least square differences (LSD) for α=0.05, and whether a post-blocking row and/or column effect was used to obtain BLUEs is reported.

|  | Madison | | | | |  | Danforth | | | |  |
| --- | --- | --- | --- | --- | --- | --- | --- | --- | --- | --- | --- |
| **Genotype** | 2018 | 2019 | 2020 | 2021 | 4-yr |  | 2018 | 2019 | 2021 | 3-yr | Overall |
| **7.02** | 330 | 367 | 384 | 380 | 368 |  | 363 | 344 | 373 | 361 | 364 |
| **8.06** | 332 | 369 | 391 | 364 | 368 |  | 374* | 368* | 386* | 376* | 371* |
| **9.1** | 314 | 372 | 375 | 357 | 356 |  | 368 | 358 | 380 | 368 | 359 |
| **11.03** | 336 | 372 | 385 | 374 | 368 |  | 381* | 367* | 383 | 377* | 369* |
| **12.05** | 342* | 366 | 387 | 374 | 370 |  | 361 | 352 | 388* | 367 | 369* |
| **47.04** | 334 | 362 | 388 | 377 | 369 |  | 372 | 358 | 378 | 369 | 368 |
| **54.08** | 338 | 364 | 388 | 380 | 368 |  | 365 | 347 | 379 | 362 | 366 |
| **138.04** | 310 | 355 | 374 | 371 | 357 |  | 368 | 359 | 369 | 366 | 358 |
| **140.05** | 348* | 377* | 382 | 382 | 376* |  | 380* | 367* | 382 | 376* | 374* |
| **145.06** | 331 | 379* | 384 | 380 | 370 |  | 366 | 363 | 368 | 365 | 365 |
| **174.01** | 351* | 384* | 398* | 385* | 379* |  | 375* | 366* | 386* | 375* | 376* |
| **183.05** | 327 | 357 | 380 | 375 | 360 |  | 369 | 356 | 366 | 364 | 358 |
| **199.09** | 327 | 381 | 388 | 381 | 372* |  | 362 | 362 | 376 | 365 | 368 |
| **202.07** | 341* | 373* | 380 | 374 | 368 |  | 367 | 359 | 372 | 366 | 365 |
| **212.08** | 347* | 379* | 387 | 376 | 374* |  | 365 | 360 | 369 | 366 | 368 |
| **260.06** | 332 | 357 | 387 | 371 | 368 |  | 377 | 359 | 378 | 372 | 367 |
| **Arapahoe** | 352* | 371 | 387 | 386* | 376* |  | 356 | 351 | 373 | 360 | 368 |
| **Warthog** | 355* | 371 | 381 | 388* | 377* |  | 354 | 347 | 383 | 370 | 372* |
| **Trial mean** | 340 | 368 | 384 | 376 | 369 |  | 368 | 357 | 377 | 368 | 367 |
| **Trial S.E.** | 1.4 | 0.9 | 0.5 | 0.4 | 0.4 |  | 0.7 | 0.4 | 0.2 | 0.2 | 0.3 |
| **Trial LSD** | 13.9 | 8.2 | 4.4 | 3.7 | 7.5 |  | 7.3 | 3.5 | 2.4 | 3.8 | 7.4 |
| **Row** | Yes | Yes | No | No | Yes |  | No | No | Yes | No | No |
| **Col** | Yes | Yes | Yes | Yes | Yes |  | No | No | Yes | No | No |
| **H^2^** | 0.62 | 0.81 | 0.97 | 0.91 | 0.70 |  | 0.72 | 0.94 | 0.99 | 0.89 | 0.87 |

Genotypes that are not significantly different (α=0.05) from the highest test weight genotype in the trial are marked with a star.

**Supplementary file 3.** Best linear unbiased estimates (BLUEs) of plant height (cm) and heading date (in Julian days) of hard red winter wheat breeding lines and commercial checks evaluated in multiple environments in three locations (Madison, Spooner, and Danforth) and five years (2017-2021). Trial mean, standard error (S.E.), heritability (H^2^), Fisher’s least square differences (LSD) for α=0.05, and whether a post-blocking row and/or column effect was used to obtain BLUEs is reported.

|  | Plant height (cm) | | | | | | | | | | | | |  | Heading date (day) | | | | | | | |
| --- | --- | --- | --- | --- | --- | --- | --- | --- | --- | --- | --- | --- | --- | --- | --- | --- | --- | --- | --- | --- | --- | --- |
|  | Wisconsin | | | | | | Spooner | | | Illinois | | | |  | Wisconsin | | | | |  | Spooner | |
| **Genotype** | 2017 | 2018 | 2019 | 2020 | 2021 | 5-yr |  | 2021 |  | 2018 | 2019 | 2021 | 3-yr |  | 2017 | 2018 | 2019 | 2020 | 2021 | 5-yr |  | 2021 |
| **7.02** | 99 | 87 | 118 | 101 | 116 | 106 |  | 90 |  | 81 | 118 | 95 | 93 |  | 154 | 157 | 158 | 155 | 145 | 153 |  | 157 |
| **8.06** | 114 | 105 | 136 | 115 | 123 | 122 |  | 95 |  | 0 | 136 | 114 | 104* |  | 154 | 160 | 158 | 159* | 147 | 156 |  | 158 |
| **9.1** | 108 | 111 | 127 | 112 | 121 | 117 |  | 90 |  | 94 | 127 | 116 | 105* |  | 154 | 158 | 151 | 159* | 148 | 154 |  | 158 |
| **11.03** | 126* | 128 | 144 | 130 | 123 | 131* |  | 98 |  | 100 | 144 | 111 | 104* |  | 158 | 162* | 151 | 162 | 153 | 157 |  | 159 |
| **12.05** | 114 | 97 | 113 | 101 | 117 | 108 |  | 88 |  | 86 | 113 | 104 | 95 |  | 150 | 153 | 157 | 156 | 144 | 151 |  | 155 |
| **47.04** | 110 | 102 | 112 | 99 | 100 | 103 |  | 78 |  | 83 | 112 | 96 | 89 |  | 154 | 158 | 156 | 158 | 145 | 153 |  | 158 |
| **54.08** | 99 | 96 | 115 | 110 | 117 | 108 |  | 102* |  | 81 | 136 | 119* | 100 |  | 152 | 156 | 158 | 158 | 145 | 154 |  | 155 |
| **138.04** | 110 | 101 | 114 | 102 | 114 | 109 |  | 87 |  | 82 | 114 | 103 | 92 |  | 160* | 163* | 151 | 160* | 147 | 156 |  | 159* |
| **140.05** | 97 | 103 | 126 | 102 | 123* | 111 |  | 90 |  | 86 | 126 | 107 | 95 |  | 153 | 157 | 158 | 158 | 147 | 155 |  | 157 |
| **145.06** | 116 | 115 | 125 | 95 | 119* | 117 |  | 90 |  | 90 | 125 | 108 | 99 |  | 157 | 155 | 162 | 158 | 148 | 156 |  | 158 |
| **174.01** | 117 | 108 | 126 | 110 | 117 | 117 |  | 93 |  | 95 | 126 | 113 | 104* |  | 147 | 160 | 156 | 156 | 145 | 151 |  | 154 |
| **183.05** | 105 | 109 | 130 | 105 | 111 | 111 |  | 85 |  | 85 | 130 | 100 | 94 |  | 156 | 158 | 151 | 160* | 154* | 157 |  | 159* |
| **199.09** | 108 | 104 | 126 | 110 | 112 | 112 |  | 88 |  | 91 | 126 | 108 | 99 |  | 157 | 156 | 154 | 159 | 148 | 154 |  | 157 |
| **202.07** | 118 | 112 | 134 | 114 | 116 | 119 |  | 87 |  | 94 | 134 | 110 | 102* |  | 157 | 162* | 162* | 160* | 153 | 158* |  | 159 |
| **212.08** | 123* | 114 | 128 | 103 | 111 | 114 |  | 92 |  | 91 | 128 | 109 | 101 |  | 153 | 161 | 162* | 158 | 147 | 156 |  | 157 |
| **260.06** | 110 | 110 | 129 | 107 | 119* | 116 |  | 90 |  | 92 | 129 | 108 | 98 |  | 157 | 160 | 151 | 159* | 152 | 156 |  | 158 |
| ‘Arapahoe’ | 104 | 110 | 112 | 108 | 104 | 110 |  | 77 |  | 77 | 112 | 98 | 88 |  | 147 | 154 | 158 | 156 | 146 | 152 |  | 155 |
| ‘Warthog’ | 100 | 98 | 116 | 96 | 106 | 104 |  | 88 |  | 81 | 116 | 92 | 87 |  | 157 | 156 | 160 | 158 | 145 | 153 |  | 157 |
| **Trial mean** | 109 | 106 | 124 | 107 | 115 | 113 |  | 89 |  | 89 | 123 | 106 | 97 |  | 154 | 157 | 156 | 158 | 148 | 155 |  | 157 |
| **Trial S.E.** | 0.49 | 1.2 | 0.6 | 1.5 | 0.6 | 0.2 |  | 0.4 |  | 0.6 | 1 | 0.3 | 0.2 |  | 1 | 0.2 | 0.4 | 0.3 | 0.1 | 0.04 |  | 0.1 |
| **Trial LSD** | 4.85 | 11.4 | 5.3 | 11.6 | 5.9 | 5.5 |  | 3 |  | 5.3 | 8.5 | 3.1 | 4.3 |  | 0.8 | 1.4 | 3.5 | 2.6 | 0.9 | 1.0 |  | 0.5 |
| **H^2^** | 0.85 | 0.84 | 0.96 | 0.87 | 0.85 | 0.87 |  | 0.87 |  | 0.87 | 0.79 | 0.97 | 0.93 |  | 0.99 | 0.95 | 0.99 | 0.84 | 0.96 | 0.47 |  | 0.94 |
| **Row** | No | Yes | No | No | Yes | Yes |  | No |  | No | No | No | No |  | No | Yes | No | No | No | Yes |  | No |
| **Col** | No | No | No | Yes | Yes | Yes |  | No |  | No | No | No | No |  | No | No | No | Yes | No | Yes |  | No |

Genotypes that are not significantly different (α=0.05) from the highest plat height or earliest plant height genotype in the trial are marked with a star.

**Supplementary file 4.** Best linear unbiased estimates (BLUEs) of ash content (%) and falling number (in seconds) of hard red winter wheat breeding lines and commercial checks evaluated in multiple environments in two locations (Danforth and Madison) and three years (2019-2021). Trial mean, standard error (S.E.), heritability (H^2^), Fisher’s least square differences (LSD) for α=0.05, and whether a post-blocking row and/or column effect was used to obtain BLUEs is reported.

|  | Ash (%) | | | | | | | |  | Falling Number (s) | | | | | | | | | |
| --- | --- | --- | --- | --- | --- | --- | --- | --- | --- | --- | --- | --- | --- | --- | --- | --- | --- | --- | --- |
|  | Danforth | | |  | Madison | | | Overall |  | Danforth | | |  |  | Madison | | |  |  |
| **Genotype** | 2020 | 20211 | 2-yr |  | 2020 | 2021 | 2-yr |  |  | 2019 | 2020 | 2021 | 3-yr |  | 2019 | 2020 | 2021 | 3-yr | Overall |
| **7.02** | - | 1.7* | 1.7 |  | 1.7 | 1.7 | 1.7 | 1.7 |  | 361 | - | 225 | 312 |  | 369 | 354 | 281 | 334 | 322 |
| **8.06** | 1.8 | 1.6* | 1.7 |  | 1.6* | 1.7 | 1.6 | 1.7 |  | 351 | 346 | 271 | 322 |  | 370 | 362 | 315 | 348 | 335 |
| **9.1** | 1.7 | 1.6* | 1.7 |  | 1.7 | 1.9* | 1.8 | 1.7 |  | 363 | 316 | 201 | 294 |  | 364 | 328 | 312 | 341 | 317 |
| **11.03** | 1.6* | 1.6* | 1.6 |  | 1.7 | 1.7 | 1.7 | 1.6 |  | 363 | 374 | 247 | 328 |  | 399 | 358 | 322* | 365 | 346 |
| **12.05** | 1.7 | 1.7* | 1.7 |  | 1.7 | 1.6 | 1.7 | 1.7 |  | 311 | 359 | 285 | 318 |  | 356 | 381 | 305 | 348 | 333 |
| **47.04** | 1.8 | 1.7* | 1.7 |  | 1.7 | 1.8* | 1.7 | 1.7 |  | 376* | 365 | 202 | 313 |  | 365 | 332 | 275 | 323 | 323 |
| **54.08** | 1.8 | 1.6* | 1.7 |  | 1.8 | 1.7 | 1.7 | 1.7 |  | 332 | 357 | 214 | 301 |  | 352 | 335 | 242 | 305 | 303 |
| **138.04** | 1.8 | 1.9 | 1.8 |  | 1.9 | 1.8* | 1.9* | 1.8 |  | - | 369 | 273 | 351* |  | 360 | 359 | 304 | 342 | 342 |
| **140.05** | 1.8 | 1.9 | 1.9* |  | 2.0 | 1.8* | 1.9* | 1.9* |  | 400* | 365 | 289 | 351* |  | 350 | 379* | 307 | 338 | 345 |
| **145.06** | - | 1.7* | 1.7 |  | 1.8 | 1.8* | 1.8 | 1.8 |  | 390* | - | 275 | 350* |  | 485* | 319 | 310 | 359 | 361* |
| **174.01** | 1.6* | 1.7* | 1.6 |  | 1.7 | 1.7 | 1.7 | 1.7 |  | 403* | 389 | 276 | 356 |  | 396 | 377* | 299 | 356 | 356 |
| **183.05** | 1.7 | 1.9 | 1.8 |  | 2.0 | 1.9* | 1.9* | 1.9* |  | 334 | 349 | 318 | 334 |  | 397 | 337 | 335 | 377 | 344 |
| **199.09** | 1.7 | 1.8 | 1.7 |  | 1.7 | 1.8* | 1.7 | 1.7 |  | 364 | 373 | 206 | 314 |  | 495 | 335 | 301 | 438* | 346 |
| **202.07** | 1.7 | 1.8 | 1.7 |  | 1.7 | 1.7 | 1.7 | 1.7 |  | 360 | 363 | 254 | 326 |  | 370 | 347 | 311 | 343 | 334 |
| **212.08** | 1.8 | 1.8 | 1.8 |  | 1.8 | 1.7 | 1.8 | 1.8 |  | 378* | 375 | 199 | 317 |  | 396 | 354 | 267 | 337 | 328 |
| **260.06** | 1.8 | 1.8 | 1.8 |  | 1.8 | 1.9* | 1.8 | 1.8 |  | 354 | 334 | 302 | 331 |  | 340 | 350 | 297 | 331 | 332 |
| ‘Arapahoe’ | 1.7 | 1.9 | 1.8 |  | 1.8 | 1.6 | 1.8 | 1.8 |  | 362 | 364 | 164 | 295 |  | 461 | 337 | 335* | 377 | 336 |
| ‘Warthog’ | 2.0 | 1.9 | 1.9* |  | 1.9 | 1.8* | 1.9* | 1.9* |  | 364 | 382 | 383* | 368** |  | 491* | 390* | 324* | 401 | 382* |
| **Trial mean** | 1.8 | 1.8 | 1.8 |  | 1.8 | 1.7 | 1.8 | 1.8 |  | 363 | 361 | 255 | 327 |  | 395 | 352 | 302 | 354 | 338 |
| **Trial S.E.** | 0.004 | 0.01 | 0.006 |  | 0.01 | 0.008 | 0.007 | 0.005 |  | 3.3 | 1.6 | 2.2 | 1.7 |  | 1.9 | 1.7 | 2.7 | 1.7 | 1.8 |
| **LSD** | 0.039 | 0.050 | 0.100 |  | 0.050 | 0.052 | 0.056 | 0.050 |  | 29.1 | 14.2 | 17.0 | 20.0 |  | 19.0 | 16.8 | 18.2 | 20.0 | 21.0 |
| **Row** | No | Yes | No |  | No | No | No | No |  | No | No | No | No |  | No | No | No | No | No |
| **Col** | No | No | No |  | Yes | Yes | No | No |  | No | No | No | No |  | No | No | No | No | No |
| **H^2^** | 0.94 | 0.91 | 0.70 |  | 0.87 | 0.82 | 0.71 | 0.87 |  | 0.47 | 0.85 | 0.96 | 0.16 |  | 0.94 | 0.93 | 0.83 | 0.47 | 0.57 |

Genotypes that are not significantly different (α=0.05) from the highest ash content or falling number value genotype in the trial are marked with a star.

**Supplementary file 5.** Best linear unbiased estimates (BLUEs) of protein content (%) of hard red winter wheat breeding lines and commercial checks evaluated in multiple environments in two locations (Danforth and Madison) and three years (i.e. 2019-2021). Trial mean, standard error (S.E.), heritability (H^2^), Fisher’s least square differences (LSD) for α=0.05, and whether a post-blocking row and/or column effect was used to obtain BLUEs is reported.

|  | Danforth | | | |  | Madison | | | |  |
| --- | --- | --- | --- | --- | --- | --- | --- | --- | --- | --- |
| **Genotype** | 2019 | 2020 | 2021 | 3-yr |  | 2019 | 2020 | 2021 | 3-yr | Overall |
| **7.02** | 9.6 | - | 9.8 | 9.4 |  | 11.9 | 10.5 | 12.2 | 11.5 | 10.4 |
| **8.06** | 9.5 | 8.9 | 10.6 | 9.8 |  | 12.8 | 10.8 | 13.2 | 12.0 | 11.0 |
| **9.1** | 10.2 | 8.8 | 9.7 | 9.4 |  | 12.4 | 10.7 | 12.6 | 12.0 | 10.7 |
| **11.03** | 9.3 | 9.1 | 10.1 | 9.8 |  | 14.0 | 12.2* | 13.1 | 13.0* | 11.4 |
| **12.05** | 9.7 | 10* | 10.6 | 10.0 |  | 12.3 | 11.5 | 12.5 | 12.0 | 11.0 |
| **47.04** | 9.2 | 8.7 | 10.0 | 9.5 |  | 12.1 | 10.4 | 11.6 | 11.2 | 10.4 |
| **54.08** | 9.3 | 9.0 | 10.1 | 9.4 |  | 12.4 | 11.0 | 12.3 | 11.9 | 10.7 |
| **138.04** | 9.1 | 9.1 | 10 | 9.4 |  | 12.7 | 10.9 | 12.5 | 11.9 | 10.7 |
| **140.05** | 10.7* | 9.9* | 11.0* | 10.5* |  | 14.8* | 12.3* | 12.6 | 13.3* | 11.9* |
| **145.06** | 9.0 | - | 9.6 | 9.8 |  | 11.9 | 10.4 | 12.4 | 11.7 | 10.7 |
| **174.01** | 9.5 | 9.3 | 10.5 | 9.6 |  | 12.2 | 10.7 | 12.3 | 11.7 | 10.7 |
| **183.05** | 8.8 | 8.8 | 9.7 | 9.2 |  | 12.4 | 10.7 | 12.8* | 12.0 | 10.6 |
| **199.09** | 9.0 | 8.6 | 9.8 | 9.0 |  | 13.4 | 10.7 | 12.8 | 12.3 | 10.6 |
| **202.07** | 8.5 | 8.7 | 9.0 | 8.8 |  | 11.7 | 10.0 | 11.8 | 11.1 | 10.0 |
| **212.08** | 9.2 | 8.9 | 10.1 | 9.5 |  | 12.9 | 11.2 | 13.3 | 12.5 | 11.0 |
| **260.06** | 8.9 | 8.7 | 9.3 | 8.9 |  | 11.7 | 10.3 | 12.0 | 11.4 | 10.1 |
| ‘Arapahoe’ | 8.6 | 8.9 | 10.9* | 9.5 |  | 12.1 | 10.0 | 10.9 | 11.4 | 10.5 |
| ‘Warthog’ | 9.0 | 8.8 | 9.5 | 8.9 |  | 12.5 | 10.0 | 11.3 | 11.2 | 10.1 |
| **Trial mean** | 9.3 | 9.0 | 10.0 | 9.5 |  | 12.6 | 10.8 | 12.3 | 11.9 | 10.7 |
| **Trial S.E.** | 0.0004 | 0.02 | 0.03 | 0.0 |  | 0.0003 | 0.02 | 0.08 | 0.02 | 0.02 |
| **LSD** | 0.0034 | 0.17 | 0.24 | 0.4 |  | 0.0032 | 0.22 | 0.52 | 0.39 | 0.38 |
| **Row** | No | No | No | No |  | No | No | No | No | No |
| **Col** | No | No | No | No |  | Yes | Yes | Yes | No | No |
| **H^2^** | 0.76 | 0.96 | 0.93 | 0.78 |  | 0.94 | 0.96 | 0.86 | 0.90 | 0.86 |

Genotypes that are not significantly different (α=0.05) from the highest protein content genotype in the trial are marked with a star.

**Supplementary file 6.** GGE biplot representation of 16 hard red winter wheat genotypes evaluated in multi-environment trials for grain yield (kg ha^-1^) for Danforth (2017-2021), Madison (2017-2021), and Spooner (2021). Wining genotypes on each mega environment are shown in bold, environments are represented in black, and genotypes in blue.


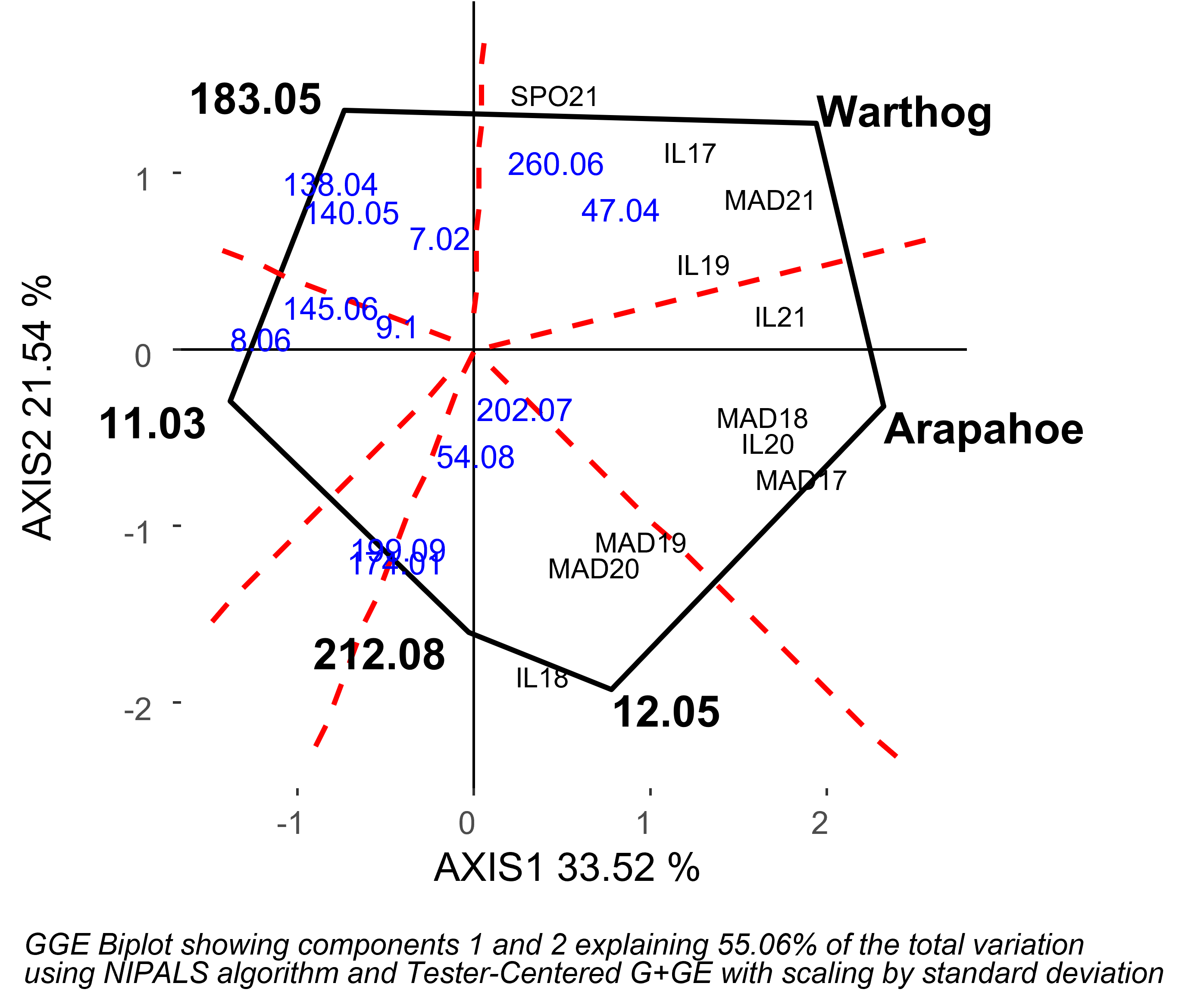


**Supplementary file 7.** DON content (ppm) performance of hard red winter wheat breeding lines and commercial check (i.e. Warthog) evaluated in multiple in two on-farm locations in 2020 (i.e. Illinois and Wisconsin) and 2021 (i.e. Wisconsin).

|  | Ridgeway, WI | |  | Freeville, NY |
| --- | --- | --- | --- | --- |
| Genotype | 2020 | 2021 |  | 2020 |
| 212.08 | - | <0.3 |  | - |
| 140.05 | 1.2 | 1.1 |  | - |
| 174.01 | 0.6 | - |  | 0.8 |
| 202.07 | - | <0.3 |  | - |
| 47.04 | 0.6 | 0.6 |  | 0.5 |
| 260.06 | 0.6 | 0.4 |  | 0.7 |
| ‘Warthog’ | 0.7 | 1.3 |  | 0.8 |
